# Hybrid modeling for in silico optimization of a dynamic perfusion cell culture process

**DOI:** 10.1002/btpr.3503

**Published:** 2024-09-18

**Authors:** Piyush Agarwal, Chris McCready, Say Kong Ng, Jake Chng Ng, Jeroen van de Laar, Maarten Pennings, Gerben Zijlstra

**Affiliations:** ^1^ Sartorius Corporate Research Oakville Ontario Canada; ^2^ Bioprocessing Technology Institute (BTI) A*STAR Biopolis Way Singapore; ^3^ BiosanaPharma Leiden Netherlands; ^4^ FMBT Marketing, Sartorius Netherlands Amersfoort Netherlands

**Keywords:** CHO cells, digital twin, hybrid models, modeling, optimization, perfusion

## Abstract

The bio‐pharmaceutical industry heavily relies on mammalian cells for the production of bio‐therapeutic proteins. The complexity of implementing and high cost‐of‐goods of these processes are currently limiting more widespread patient access. This is driving efforts to enhance cell culture productivity and cost reduction. Upstream process intensification (PI), using perfusion approaches in the seed train and/or the main bioreactor, has shown substantial promise to enhance productivity. However, developing optimal process conditions for perfusion‐based processes remain challenging due to resource and time constraints. Model‐based optimization offers a solution by systematically screening process parameters like temperature, pH, and culture media to find the optimum conditions in silico. To our knowledge, this is the first experimentally validated model to explain the perfusion dynamics under different operating conditions and scales for process optimization. The hybrid model accurately describes Chinese hamster ovary (CHO) cell culture growth dynamics and a neural network model explains the production of mAb, allowing for optimization of media exchange rates. Results from six perfusion runs in Ambr® 250 demonstrated high accuracy, confirming the model's utility. Further, the implementation of dynamic media exchange rate schedule determined through model‐based optimization resulted in 50% increase in volumetric productivity. Additionally, two 5 L‐scale experiments validated the model's reliable extrapolation capabilities to large bioreactors. This approach could reduce the number of wet lab experiments needed for culture process optimization, offering a promising avenue for improving productivity, cost‐of‐goods in bio‐pharmaceutical manufacturing, in turn improving patient access to pivotal medicine.

## 1 INTRODUCTION

Chinese hamster ovary (CHO) cell lines are extensively utilized in the production of therapeutic antibodies of appropriate quality. To ensure sufficient patient access, efficient large‐scale manufacturing is required, and developing a robust and scalable culture process with high productivity is crucial. Therefore, one of the primary objectives in fed‐batch cell culture process development is to achieve a high yield.^1^, ^2^

While fed‐batch culture remains the most commonly employed method in the bio‐pharmaceutical industry due to its simplicity and robustness, substantial efforts are ongoing to improve mammalian cell culture productivity through process intensification (PI). In upstream processing, this can be achieved, among other methods, by implementing perfusion strategies in the seed train and/or the main bioreactor, as extensively described in several review papers.^3^, ^4^, ^5^ With perfusion cultures gaining in popularity, optimization of process conditions, media or suitable cell lines specific to the new demands, is essential. Such multidimensional analysis can take numerous experimental attempts, inflating costs and risks associated with development. This represents a challenge for perfusion specifically, as the extreme cell densities and continuous nature of its operation makes perfusion runs very demanding.^3^ The reason for the large number of experiments required is that process parameters such as temperature, feeding strategy, dissolved oxygen (DO), and pH are typically optimized using design‐of‐experiments (DoE)^6^ or one‐factor‐at‐a‐time (OFAT) studies.^7^ Among these optimization strategies, temperature downshifting and feeding amount adjustment are frequently employed. Temperature downshifting is generally performed during the mid to late exponential phase by switching the culture temperature from approximately 37°C to a lower range (i.e., 29–35°C). This reduction in temperature helps to reduce cell apoptosis, promotes antibody synthesis, and typically has a positive impact on integral cell density, cell viability, and specific productivity.^8^, ^9^ Additionally, feed media composition and feeding strategy are critical factors in cell culture process development.^10^, ^11^ Due to the numerous potential combinations of the process parameter values and strategies described, the experimental design space is very large. This has been addressed to a large degree by the advent of high throughput perfusion bioreactor systems, such as Ambr® HT perfusion, that have addressed this bottleneck by providing a platform for automated high throughput perfusion experimentation.^12^ In reality, however, due to time constraints and limited resources in early‐phase projects, it is still impractical to explore the full design space with all potential process modes to identify the most optimal ones.

Therefore, in a quest to reduce time consuming and expensive experimental process optimization, considerable research efforts have been made into process modeling and in particular hybrid modeling aiming to better understand the complex dynamics of these systems. Hybrid modeling is regarded as key enabler toward in silico optimization and ultimately realizing digital twins in biopharma.^13^ Many approaches for hybrid modeling of CHO and other cell culture processes and in particular variation in growth dynamics exist in literature.^14^, ^15^, ^16^, ^17^, ^18^ Pinto et al.^14^ provide a nice compilation of hybrid modeling studies for CHO cells in Fed Batch processes. It is typical that growth and death rates are connected to concentration of measured process conditions and metabolites such as in Maria (2020),^15^ where growth rate is a function of concentration of substrate and growth inhibiting compounds. Narayanan et al.^19^ demonstrate various implementations of hybrid modeling strategies to estimate growth and death rates. These strategies are successfully applied to fed‐batch type cell culture processes. In perfusion processes, it is shown in Richelle et al.^20^ that a significant driver of growth dynamics is the removal of inhibitory and toxic materials. Using some basic assumptions, accumulation of these materials can be estimated using material balance equations without the need for any additional measurements. The perfusion rate in a perfusion processes is typically selected based on (a) cell density to achieve a cell specific perfusion rate (CSPR), (b) metabolite measurements such as glucose, or (c) a representative measure of cell density such as oxygen consumption.^21^ These methods are based around providing sufficient nutrients to support growth and metabolism.

Hybrid modeling in CHO cell culture involves integrating mechanistic models, which capture the underlying biological mechanisms and process kinetics, with data‐driven models that leverage experimental data to improve predictive capabilities.^18^, ^22^, ^23^, ^24^, ^25^ This integration enables a more comprehensive representation of the complex interactions occurring within the culture system. Mechanistic models typically describe cell growth dynamics and material balance of biological metabolites (substrates, amino acids, and biomaterials excreted by the cells) using mathematical equations based on biochemical principles. Here, monod‐type models are widely used to describe CHO cell culture kinetics because they capture the most important measurements such as cell density, nutrients, and metabolites while maintaining the simplicity of the equations.^26^, ^27^ On the other hand, data‐driven models utilize statistical (e.g., OPLS, SIMCA^28^) and machine learning (ML) techniques to learn patterns and relationships from experimental data to explain shifts in metabolism, such as nutrient uptake, metabolism, and product formation resulting from variation in bulk fluid conditions. By combining mechanistic and data‐driven models, hybrid modeling takes advantage of the strengths of both approaches, with mechanistic models providing a deep understanding of the underlying biological processes, while data‐driven models capture the complexity and nonlinearities of the system.^29^ This integration allows for more accurate predictions and the ability to capture system behavior under different operating conditions and perturbations. Hybrid models have also emerged as powerful tools for optimizing complex processes such as downstream chromatography. The innovative aspect lies in the reduction of computational time required for simulation and optimization, enabling rapid exploration of various design scenarios.^30^ In the realm of regenerative medicine, computational models offer invaluable insights into optimizing bioreactor processes for neotissue growth. By transitioning from intricate mechanistic models to computationally streamlined versions, as demonstrated in one study,^31^ researchers can efficiently explore optimal process conditions. Optimizing these bioreactors is a complex engineering challenge because of the non‐linear dynamics and multiple state‐variables involved, leading to a non‐convex optimization problem with numerous decision variables.^32^ In another work, particularly the hybrid rate (HR) model, it was found to offer optimal performance by balancing process knowledge and model parameters. The concept is seen as valuable for developing and testing hypotheses in complex processes like cell cultures.^19^

Therefore, in theory model‐based simulations have always had the potential to substantially reduce the experimental effort for bio‐pharmaceutical process development. But with the recent improvements in digital and experimental infrastructure, in silico modeling and process simulation have emerged as a practical tool for screening process parameters thus reducing the need for a large number of wet‐lab experiments and are increasingly adopted.^33^

In this work, the hybrid cell culture model is focused on growth dynamics being driven by accumulation of unmeasured materials rather than supply of nutrients. A shallow neural network (NN) is integrated to additionally predict the concentration of a monoclonal antibody (mAb). Predicted process conditions from a mechanistic part of the cell culture model such as predicted viable cell density, lysed cell concentration, accumulation of unknown biomaterial, pH, temperature, and working volume are used as inputs to the NN to predict specific productivity and optimize feeding policy for a perfusion process. To enable this, perfusion runs with different media exchange rates were performed for hybrid modeling. For the CHO platform presented in this study, several key process parameters can affect the productivity, longevity, viability, and product quality of the dynamic perfusion cell culture processes. Cell cycle arrest followed by cell size increase, and associated productivity increase plays a key role in cell culture performance. A set of Ambr® 250 high throughput (HT) perfusion bioreactor runs were performed to generate cell culture data, providing insights into the impact of cell culture conditions on dynamic perfusion performance and productivity. We demonstrate that the identified model predictions matched the experimental runs well for a wide range of different operating conditions such as glucose set‐point, culture termination period, choice of filter, and perfusion rate. Two more Ambr® runs were carried out where they were operated at different conditions than the baseline runs for model validation in Sections 4.2 and 4.3. The growth model simulations successfully predicted the culture dynamics and titer for both the batches indicating high extrapolation capabilities of the identified model and adequacy to reduce the number of wet‐lab experiments required for process optimization. Additionally in Section 4.4, we demonstrate a successful experimental validation of our optimal feeding policy determined using the identified hybrid model from the reference batches for maximizing the total product collected in the harvest stream throughout the duration of the production phase. A comparison of the total product captured indicates a 50% increase as compared with the best run of the training set (from Day 4 to Day 21).

One of the primary goals of conducting Ambr® 250 experiments was to generate data that can be reliably extrapolated to larger bioreactors. However, ensuring the transferability of results requires careful evaluation and understanding of the differences between the Ambr® system and the target large‐scale bioreactor. Towards this end, we present successful simulations of 5 L perfusion runs using a model identified based on Ambr® experiments in Section 4.5.

## 2 MATERIALS AND METHODS

### 2.1 Cell line, media, and cell expansion

The cell line used was a proprietary CHO K1‐derived cell line (BiosanaPharma), producing an IgG1 mAb. All cultures were started from a research cell bank, derived from the MCB established in 2015. Starting with a research cell bank at 1E7 cells, cells were expanded in shake flasks at 37°C in 5% CO_2_ at 120 rpm and a 25 mm pitch. The culture was passaged every 3–4 days with a seeding density of 4E5 cells/mL and around 20% working volume to total volume. To inoculate the Ambr® perfusion experiments, cells were expanded to inoculate 6 × Ambr® 250 vessels with a target seeding density of 25E5 cells/mL and a working volume of 223 mL. To inoculate the 5 L bioreactors with a target seeding density of 25 E5 cells/mL, in the final passage before reactor inoculation, 2 × 1 L shake flasks with 200 mL working volume, were seeded at 5–6 E5 cells/mL. Media used during cell expansion was IS CHO‐CD G10.7 (Fujifilm), supplemented with 6 mM L‐glutamine (Sigma Aldrich) and 2.2 g/L NaHCO3 (Sigma Aldrich). During cell expansion cell density and viability were measured with a ViCell XR cell counter (Beckman).

### 2.2 Perfusion cell cultures in Ambr® 250 HT perfusion

This study was performed in a 12‐way Ambr® 250 HT perfusion (Sartorius) with an integrated Analysis module for pH measurement and integrated NovaFlex 2 for at‐line cell density, metabolite and physical parameter measurements, allowing feedback loops for advanced process control. In this study, 2 series of 3 Ambr® 250 perfusion vessels with similar settings were used in TFF mode, using either the default MIDIKROS 0.2 μm, PES filters (Repligen) or Microza 0.2 μm, PVDF (Asahi) filters. For each filter type, three different perfusion rates were used. The perfusion strategy was based on biomass volume. This was calculated by multiplying viable cell number with average cell volume and was used to adjust the perfusion rate (i.e., biomass specific perfusion rate [BSPR]) during the production phase. The medium used in the perfusion bioreactor was customized media from Fujifilm, with 4 mM L‐glutamine. The growth phase of the culture was controlled at 37°C, pH at 7.05 ± 0.05 and 50% DO. The production phase was at 34°C, pH 6.95 ± 0.05 and 50% DO. pH was controlled with CO_2_ sparging through Ring sparger and 1 M sodium carbonate addition. FoamAway (Thermofisher) was added automatically via foam sensor. More details on the settings can be found in Table 1. Further two more Ambr® 250 runs were carried with operating conditions outside of the training set to test the predictions of the identified model (batches V01 and V02) and the details can be found in Table 2.

**TABLE 1:** Ambr® 250 process parameters.

Parameter	Training batches			Validation batches		
	B01	B04	B05	B02	B03	B06
Culture volume	200 mL (stirred volume)					
Inoculation cell density	2.5 E6 cells/mL					
Production cell density	60 E6 cells/mL					
Growth phase temperature	37°C					
Production phase temperature	34°C					
Criteria for shifting to production temperature	When VCD >50 E6 cells/mL					
Glucose set‐point	D0‐19: 3 g/L D20‐25: 6 g/L	D0‐13: 3 g/L D14‐20: 6 g/L	3 g/L	3 g/L	3 g/L	D0‐19: 3 g/L D20‐25: 6 g/L
Culture termination	D25	D20	D25	D25	D20	D25
Production perfusion rate	6.5 mL/cm^3^/day	8 mL/cm^3^/day	12 mL/cm^3^/day	6.5 mL/cm^3^/day	8 mL/cm^3^/day	12 mL/cm^3^/day
Hollow fiber filter	Microza	MIDIKROS	Microza	MIDIKROS	Microza	MIDIKROS

**TABLE 2:** Ambr® 250 process parameters for test runs.

Parameter	Test batches	
	V01	V02
Culture volume	200 mL (stirred volume); 223 mL (volume inclusive of filter column)	
Inoculation cell density	2.5 E6 cells/mL	
Production cell density	65 E6 cells/mL	
Growth phase temperature	37°C	
Production phase temperature	34°C	
Criteria for shifting to production temperature	When VCD >60 E6 cells/mL	
Glucose set‐point	3 g/L	3 g/L
Culture termination	D17	D17
Production perfusion rate	9 mL/cm^3^/day	8 mL/cm^3^/day
Hollow fiber filter	MIDIKROS	MIDIKROS

### 2.3 Perfusion cell cultures in 5 L benchtop bioreactor

Several perfusion process runs were performed in 5 L benchtop bioreactors as an intermediate‐scale process characterization tool. A number of these runs were selected for model verification of the Hybrid model developed with the Ambr runs. Main differences between these runs include the CSPR (cell‐specific perfusion rate) range in the production phase resulting from feeding strategies to maintain Glucose concentration constant. The perfusion cultures were performed in 5 L Univessel bioreactor (Sartorius) with a working volume of 5.0 L and equipped with a TFF loop with a low shear centrifugal recirculation pump (Levitronix) and controlled by a Biostat B‐DCU with MFCS Supervisory control using a customized Perfusion Recipe (Sartorius). For cell retention Microza TFF filters were used (Asahi). A capacitance probe (Aber) was used to maintain cell density by automated cell bleeding. The culture was inoculated at 2.5 × 10^6^ cells/mL. The perfusion medium was a customized media from Fujifilm, with 4 mM of L‐glutamine. The growth phase of the culture was controlled at 37°C, pH at 7.05 ± 0.05 and 50% DO. The production phase was at 34°C, pH 6.95 ± 0.05 and 50% DO. pH was controlled with CO_2_ sparging through Ring sparger and 1 M Sodium Carbonate addition. DO was controlled with O_2_ and Air sparging through micro sparger. Mixing was through increasing the stir rate from 140 to 220 rpm depending on viable cell density. Lastly, FoamAway (Thermofisher) was added automatically via foam sensor.

### 2.4 Analytical methods

Cell densities, viabilities, and cell size distribution were measured online for the 5 L Benchtop bioreactors or at‐line for the Ambr® 250 HT perfusion using NovaFlex 2. Metabolites (including Glucose, Lactate, Glutamine, and Ammonia) and physical parameters (incl. pH, pCO_2_ and osmolarity) were measured using NovaFlex 2 and the Ambr® Analysis module for pH (Sartorius). Product titer was measured both in the bioreactor supernatants, as well as in the TFF filter permeates using analytical Protein A chromatography (Dionex Ultimate 3000 UHPLC).

## 3 HYBRID STATE SPACE MODEL

The objective of mathematical modeling methodology is to build a generalized model with a single set of kinetic coefficients representing all phases of a batch or fed‐batch including exponential growth, stationary, and death phases as well as complex processes such as perfusion where the operating mode transitions from an initial intensified growth phase followed by a pseudo‐steady‐state perfusion phase. This contrasts with approaches that identify separate kinetic parameters as the cell culture progresses through different phases or operating modes.^34^

### 3.1 Perfusion cell culture modeling principles

CHO cells are typically cultivated in a bioreactor with controlled environmental conditions. CHO cell culture population can be divided into three subgroups: living ($X_{v}$), dead ($X_{d}$), and lysed ($X_{l}$) cells. The cell growth rate is inhibited by the accumulation of metabolic byproducts (here represented by a catch‐all “biomaterial” variable $\Phi_{b}$). The cell death rate is accelerated by the accumulation of toxic materials. In (Richelle et al.^20^), the lysed cell concentration is used to explain the increase in death rate due to accumulated toxins. A bioreactor can be operated in different modes by acting on the inlet (feeding medium addition, $F_{f}$) and outlet ($F_{\text{out}}$) flow rates:

- Batch mode: all substrates are added at the beginning of the culture and nothing is added ($F_{f}=0$) or removed ($F_{\text{out}}=0$) from the bioreactor afterward;
- Fed‐batch mode: the bioreactor is fed with culture medium throughout the duration of the run ($F_{f}\neq 0$), while the outflow remains null ($F_{\text{out}}=0$);
- Perfusion mode: the bioreactor is fed continuously ($F_{f}\neq 0$) and the culture medium is continuously removed ($F_{\text{out}}\neq 0$).

**FIGURE 1:**
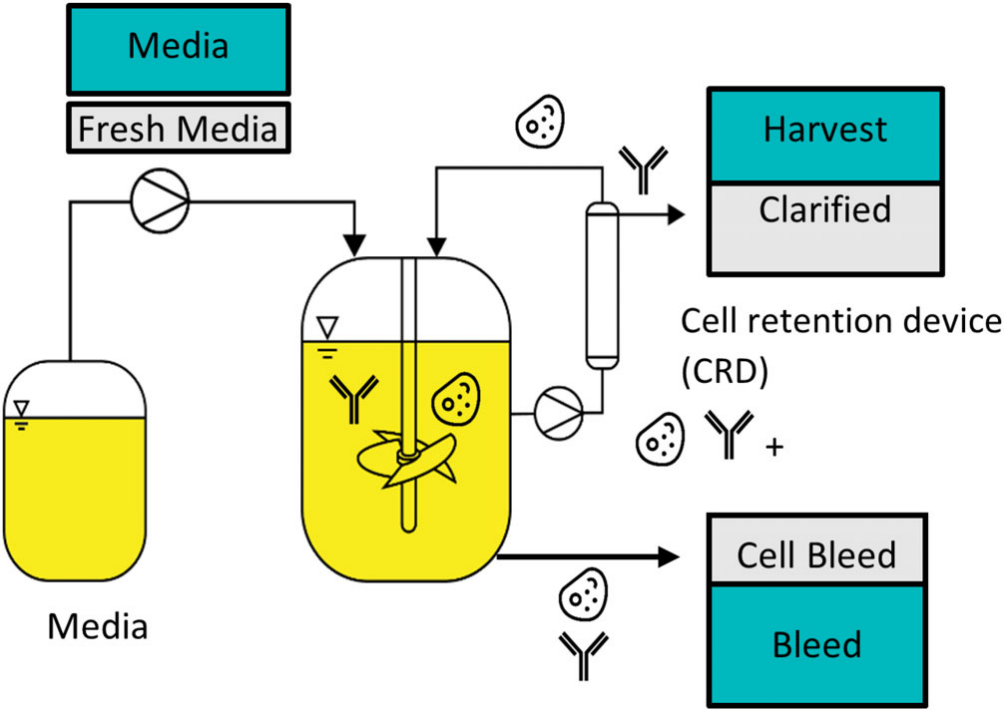
Schematic of a perfusion bioreactor.

In this work, we will focus on continuous perfusion mode of operation only for optimization. Perfusion is a type of continuous operation where the cell culture is continuously fed with fresh medium ($F_{f}$) while the outlet flow $F_{\text{out}}$ is composed of a harvest ($F_{h}$) and a “bleed” ($F_{b}$) stream that is removed to maintain a desired viable cell density and volume (Figure 1). To this end, the harvest stream ($F_{h}$) is firstly directed through a cell retention device (CRD) that will separate the living ($X_{v}$) and dead ($X_{d}$) cells from the used media containing lysed cells ($X_{l}$) and metabolic byproducts ($\Phi_{b}$). The living ($X_{v}$) and dead ($X_{d}$) cells are then re‐injected in the bioreactor while the cell‐free stream is collected for further purification of the drug product. The “bleed” outflow ($F_{b}$), presenting the same composition as the bioreactor is used to remove solids, such as viable and dead cells, and other materials that do not pass through the CRD, to maintain the culture at a desired state (i.e., maintain the concentration of living cells within the bioreactor constant).

### 3.2 Growth model

The growth model consists of differential equations describing the dynamic material balance of viable, dead and lysed cells. Data‐driven models are used to describe the specific productivity and consumption and/or secretion rates of metabolites such as nutrients or measured biological byproducts such as ammonia. However, in this study, it was observed that no metabolite was growth‐inhibiting or limiting. Hence, consumption rates were not modeled. Using this formalism, the dynamic of the 3 cell population subgroups ($X_{v}$, $X_{d}$ and $X_{l}$) and the accumulation of the uncharacterized set of growth‐inhibiting biomaterials secreted by the cells ($\Phi_{b}$) can be described with the following set of ordinary differential equations:

(1) $$\frac{{\mathrm{dX}}_{v}}{\mathrm{dt}}=\mu_{\mathrm{eff}}X_{v}-\mu_{d}X_{v}-\frac{F_{b}}{V}X_{v}$$

(2) $$\frac{{\mathrm{dX}}_{d}}{\mathrm{dt}}=\mu_{d}X_{v}-\left(k_{l}+\frac{F_{b}}{V}\right)X_{d}$$

(3) $$\frac{{\mathrm{dX}}_{l}}{\mathrm{dt}}=k_{l}X_{d}-\left(\frac{F_{h}+F_{b}}{V}\right)X_{l}$$

where $X_{v}$ is the viable cell density (VCD—concentration of living cells), $X_{d}$ is the dead cell density (concentration of dead cells), $X_{l}$ is the lysed cell density (concentration of lysed cells), and $\Phi_{b}$ is a catch‐all “biomaterial” variable representing the growth‐inhibiting metabolic byproducts. $F_{b}$ is the bleeding rate, $F_{h}$ is the harvest rate (also known as permeate flow), and V is the bioreactor volume. $\mu_{\mathrm{eff}}$, $\mu_{d}$, and $k_{l}$ are the effective growth, effective death, and lysing rates, respectively.

Here, we assumed that lysed cells $X_{l}$ refer to materials that accumulate in the spent media from dead cells $X_{d}$ that have lysed and dead cells arise from living cells $X_{v}$, according to the specific death rate $\mu_{d}$. Kroll et al.^35^ showed that the lysed cells could be a direct degradation product of living cells $X_{v}$. However, we were not able, in the case of this study, to discriminate the dead and lysed cells with the analytical assays used to assess the cell viability (trypan blue exclusion test [Strober^36^]). Therefore, we decided to keep the lysed cell subpopulation as a degree of freedom in the model structure.

These mass balance equations are developed under the assumption that the bleed stream ($F_{b}$) has the same content as the bioreactor and the harvest stream ($F_{h}$) is cell‐free (ideal separation filter where the lysed cells and biomaterial pass‐through, and only viable and dead cells are retained into the bioreactor).

The effective cell growth rate ($\mu_{\mathrm{eff}}$) is typically modeled as the product of the maximum growth rate ($\mu_{\max}$), growth inhibiting factors such as lactate or ammonia, exhaustion of substrates such as glucose and influence of process conditions such as temperature or pH. A generalized form of the effective growth rate is:

(4) $$\mu_{\mathrm{eff}}=\mu_{\max}.\prod_{i=1}^{S}\;\eta_{S,i}.\prod_{i=1}^{Q}\;\eta_{Q,i}.\prod_{i=1}^{I}\;\eta_{I,i}$$

where $\eta_{s,i}$ is the contribution for the substrate variable $S_{i}$, $\eta_{Q,i}$ is the contribution for the quadratic variable $Q_{I}$ and $\eta_{I,i}$ is the contribution for the inhibitor variable $I_{i}$. The choice of the mathematical formalism used to describe each of these contributions depends on the case under study. The term “quadratic” is borrowed from DoE terminology where quadratic variables are used to describe factors that have an optimum value, while in actuality an exponential bell curve is used to fit these effects to assure stable simulations. In this work, variables such as temperature, and pH are treated as independent variables and defined as inputs and have quadratic effects on the growth (Equation 5). As such, there is no thermodynamic model to represent changes in temperature or model of base addition and other influences of pH. It is assumed the regulatory control layer provides a controllable process that maintains inputs at their set points. This decision is taken to remove scale and hardware‐dependent dynamics, producing a scale‐independent model. This also means that the hybrid state space model (HSSM) model is primarily relevant for processes which have established stable regulatory control. Given the use cases targeted for this model center around optimization and system configuration, the separation of scale‐dependent and regulatory control issues limits the scope of the HSSM to be manageable and fit for purpose.

(5) $$\eta_{Q,i}=\exp\left[-\frac{1}{25}{\left(\frac{z_{n}-\mu_{q,n}}{\theta_{q,n}}\right)}^{2}\right]$$

where $\mu_{q,n}$ is a parameter that represents the target value (i.e., the value at which maximum growth occurs) and $\theta_{q,n}$ is the “spread” of the effect.

The approach described in (Richelle et al.^20^) is used including a parameter, biomaterial ($\Phi_{b}$), which represents a collection of unmeasured materials secreted by the cells that accumulate in the spent media. Assuming a constant specific rate of production of these biomaterials and that they pass through the CRD, the material balance on $\Phi_{b}$ is:

(6) $$\frac{d\Phi_{b}}{\mathrm{dt}}=X_{v}-\left(\frac{F_{h}+F_{b}}{V}\right)\Phi_{b}$$

$\Phi_{b}$ is included in the generalized growth rate function (Equation 4) to produce:

(7) $$\mu_{\mathrm{eff}}=\mu_{\max}.\frac{1}{{\left(\frac{\Phi_{b}}{K_{I,\Phi_{b}}}\right)}^{3}+1}.\prod_{i=1}^{S}\eta_{S,i}.\prod_{i=1}^{Q}\eta_{Q,i}.\prod_{i=1}^{I}\eta_{I,i}$$

where $K_{I,\Phi_{b}}$ represents the threshold above which biomaterial concentration ($\Phi_{b}$) begins to inhibit growth rate.

As in (Richelle et al.^20^) the effective death rate, $\mu_{d}$, is dependent on a base death rate and a toxicity factor related to the accumulation of lysed cells ($X_{l}$). Functionally:

(8) $$\mu_{d}=k_{d}+k_{t}X_{l}$$

where $k_{d}$ is the primary death rate and $k_{t}$ represents the toxicity factor associated with the accumulation of lysed cells in the spent media.

Finally, the lysing process was assumed to be governed by $k_{l}$ through a first‐order rate law. This assumption was not further investigated as the lysed cell subpopulation is acting as a degree of freedom in the model structure. Tracking the material balance of viable and dead cells indicates the total cells generated and by extension the number of cells that have lysed and are no longer detectable. Dead cells are evaluated indirectly through cell viability measurement which captures the ratio between viable cells and total cells:

(9) $$\text{Viability}=\frac{X_{v}}{X_{v}+X_{d}}$$

### 3.3 Productivity model

**FIGURE 2:**
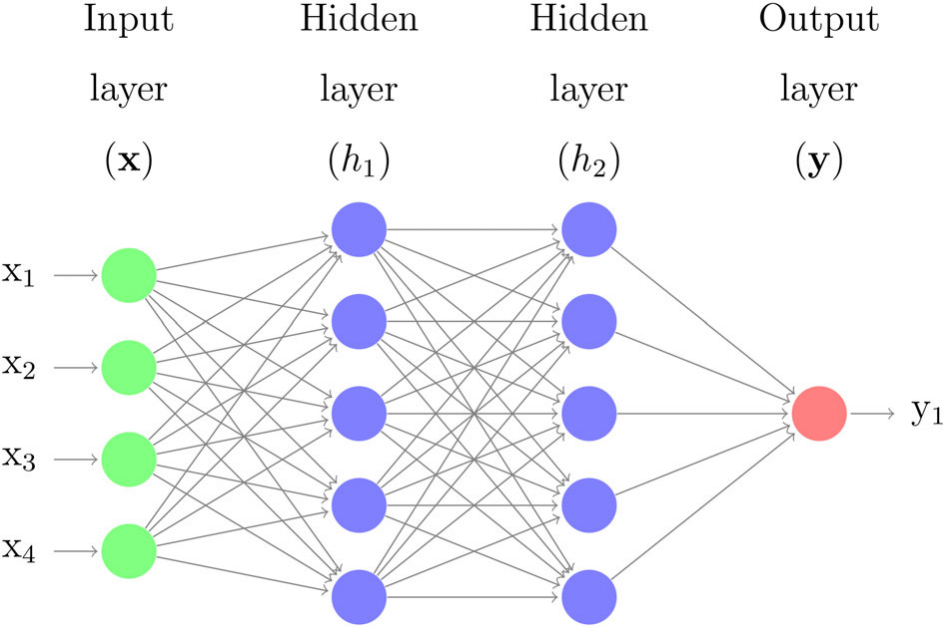
Multi‐layer perceptron neural network.

Protein production, that is, titer concentration is estimated by integrating the specific productivity $q_{p}$ into the state equation (titer) to track the accumulation of product as below:

(10) $$\frac{d\left[\mathrm{IgG}\right]}{\mathrm{dt}}=q_{p}X_{v}-\left(\frac{F_{h}+F_{b}}{V}\right)\left[\mathrm{IgG}\right]$$

where specific productivity $q_{p}$ is predicted using a multilayer perceptron neural network (MLP‐NN). An MLP is a class of feed‐forward network (also known as an artificial neural network) used for both classification and regression problems.^37^, ^38^ Since single perceptron (neuron) models are unable to explain complex nonlinear functions,^39^ hence several stacked neurons and layers are needed to approximate nonlinear complex functions. Each neuron layer is stacked one after another and connected to each neuron in the following layer. Figure 2 shows a schematic for a basic Multi‐Layer Perceptron of three layers. The network consists of three types of layers: an input, hidden, and output layer. The input layer is fed with the data available in the problem. The hidden layers are used by the network to learn the important features that are contained in the input data. The output layer provides a final response that might be a class label in problems of classification or continuous values in general forecast/prediction problems. It is important to note that there are no limitations on the number of layers or the number of neurons in a network. However, in feed‐forward networks, there are no interconnections among neurons within the same layer; thus, the data is transmitted only in the forward direction.

To summarize the figure, each neuron $h_{j}^{1}$ in the first hidden layer (*h* ^1^) is connected to each input neuron in the input layer $x=x_{1},x_{2},\ldots ,x_{n}$, each of which have specific weights associated to each input‐hidden layer connection $w_{n}=w_{1j},w_{2j},\ldots ,w_{\mathrm{nj}}$ and a bias term $\theta_{\mathrm{ij}}$. The weighted sum of the inputs is then calculated using the following formula:

(11) $$z_{j}=\sum_{i=1}^{n}\;x_{i}w_{\mathrm{ij}}+\theta_{\mathrm{ij}}$$

The value of $z_{j}$ is then passed through an activation function $f\left(z_{j}\right)$, which is the key decision‐making unit, which depending on the task to be accomplished can take different forms. Consider for instance the following “log‐sigmoid” activation function:

(12) $$f\left(z_{j}\right)=\frac{1}{1+e^{-z_{j}}}$$

then the output of $h_{j}^{1}$ is computed as

(13) $$h_{j}^{1}=\frac{1}{1+e^{-\left(\sum_{i=0}^{n}x_{i}w_{\mathrm{ij}}+\theta_{\mathrm{ij}}\right)}}$$

The output from each node in the hidden layer is computed and the resulting values are used as inputs for the following layer. The outputs from the last hidden layer are considered inputs for the output layer. NN is trained to optimize the model weights that is weights and biases by minimizing an objective function. For regression, mean square error is typically used as an objective function for training NNs.

(14) $$\min_{W,b}\mathrm{MSE}=\min_{W,b}\;\frac{1}{N}\left[{\left\|y_{s}-{\hat{y}}_{s}\right\|}_{2}^{2}\right]$$

where N is the total number of samples.

*Note*: In Equation (10), there are no sieving effects, that is, it is assumed all product passes through the CRD.

## 4 RESULTS AND DISCUSSION

This section presents the main results of this study. First, the proposed growth model is fitted to the measured VCD and cell viability for the perfusion runs. Then, a data‐driven NN model is trained and validated to estimate specific productivity. Additionally, an optimization solver is employed to develop an optimal feeding strategy with the aim of maximizing the total product collected over the 17 days following a growth phase of 4 days. Further, we demonstrate the scale validation by simulating the growth kinetics in a 5 L scale benchtop bioreactor using a model based on Ambr® 250 experiments.

### 4.1 Parameter estimation

To estimate optimal model parameters, that is, maximum growth rate: $\mu_{\max}$, specific death rate: $\mu_{d}$, toxicity rate: $k_{t}$, lysing rate: $k_{l}$, inhibition coefficient (accumulation of unknown biomaterial): $K_{I,\Phi_{b}}$ and spread coefficients for the quadratic effect of inputs, that is, pH and temperature: $\theta_{q,\mathrm{pH}}$ and $\theta_{q,\text{temp}.}$, mentioned below: (a) a set of ODE (Equations (1), (2), (3), (4), (5), (6), (7), (8), (9)) is solved using Scipy's “solve_ivp” function and VCD setpoint is taken from the measured VCD; (b) the comparison of measured bleed to bleed evaluated from the model is used in the objective function; (c) prediction errors are calculated concerning bleed and cell viability; and (d) auto‐covariance is minimized iteratively using a stochastic optimization algorithm called particle swarm optimization (PSO) algorithm as described in Adams and Warren.^40^ This is a global optimization method that has proven to be effective in the identification of the HSSM parameters. The implementation in the HSSM library has a flexible identification procedure where the number of iterations and step size are selected by user input. For these models, a two‐step procedure was used where a relatively large step size is first used to explore the parameter space to find the general location of the global minima followed by a second with a reduced step size to hone in on the optimal set.

To circumvent local minima and convergence problems, model parameters were bounded between a lower and upper bound (through experience) to constrain the space of exploration during optimization (refer Table 4). The identified parameter values based on three experimental runs are presented in Table 3.

**TABLE 3:** Estimated model parameters for each experiment separately and together.

Parameters	Batch “B01”	Batch “B04”	Batch “B05”	Batches “B01, B04, B05”
$\mu_{\max}$	0.837	0.808	0.832	0.848
$\mu_{d}$	0.01047	0.00746	0.00739	0.00921
$k_{t}$	0.03497	0.03468	0.03947	0.03514
$k_{l}$	1.55083	1.60921	1.37549	1.58991
$K_{I,\Phi_{b}}$	13.7618	16.1469	12.5970	12.7205

**TABLE 4:** Upper and lower bounds of model parameters.

Parameters	Lower bound	Upper bound
$\mu_{\max}$	0.05	1.2
$\mu_{d}$	0.001	0.05
$k_{t}$	0.000001	0.1
$k_{l}$	0.8	2
$K_{I,\Phi_{b}}$	10	250
$\theta_{q,\mathrm{pH}}$	0.000271	3.301
$\theta_{q,\text{temp}}$	0.011	0.081

**TABLE 5:** Optimized volumetric media exchange rates.

Time (days)	0	4	6	9	13	17
Exchange rate (VVD)	2.04	0.81	1.19	1.70	1.89	1.95

**TABLE 6:** Estimated parameters for both 5 L‐scale runs.

Experiment	$K_{I,\Phi_{b}}$	$k_{\mathrm{bh}}$	$k_{t}$	$k_{\mathrm{ls}}$
Run 1	12.139	0.014	0.049	0.023
Run 2	12.70	0.010	0.035	0.010

### 4.2 Model fitting

Three batches (namely B01, B04, and B05) out of six perfusion runs with different feed strategies (as shown in Figure 2) were used to model the impact of media exchange on growth dynamics by calibrating the abovementioned growth model (refer to Equations (1), (2), (3), (4), (5), (6), (7)). The set of ordinary differential equations was solved using Scipy's “solve_ivp” function in Python3^41^, ^42^ and PSO was used to estimate the optimal model parameters, that is, maximum growth rate: $\mu_{\max}$, specific death rate: $\mu_{d}$, toxic sensitivity: $k_{t}$, lysing rate: $k_{l}$, inhibition coefficient (accumulation of unknown biomaterial): $K_{I,\Phi_{b}}$, spread coefficients for the quadratic effect for inputs, that is, pH and temperature: $\theta_{q,\mathrm{pH}}\&\theta_{q,\text{temp}}$ by minimizing the autocorrelation of the residuals of predicted bleed and cell viability. The fitting and prediction performance can be evaluated in comparison to the experimental data using $R^{2}$ (Equation 16).

(15) $$\text{NMSE}=\sum_{i=X_{v},\text{Viab}}^{2}\frac{{\mathrm{MSE}}_{i}}{{\left(y_{\max,i}-y_{\min,i}\right)}^{2}}$$

(16) $$R^{2}=1-\frac{\sum_{i}^{n}{\left(y_{i}-{\hat{y}}_{i}\right)}^{2}}{\sum_{i}^{n}{\left(y_{i}-\bar{y}\right)}^{2}}$$

**FIGURE 3:**
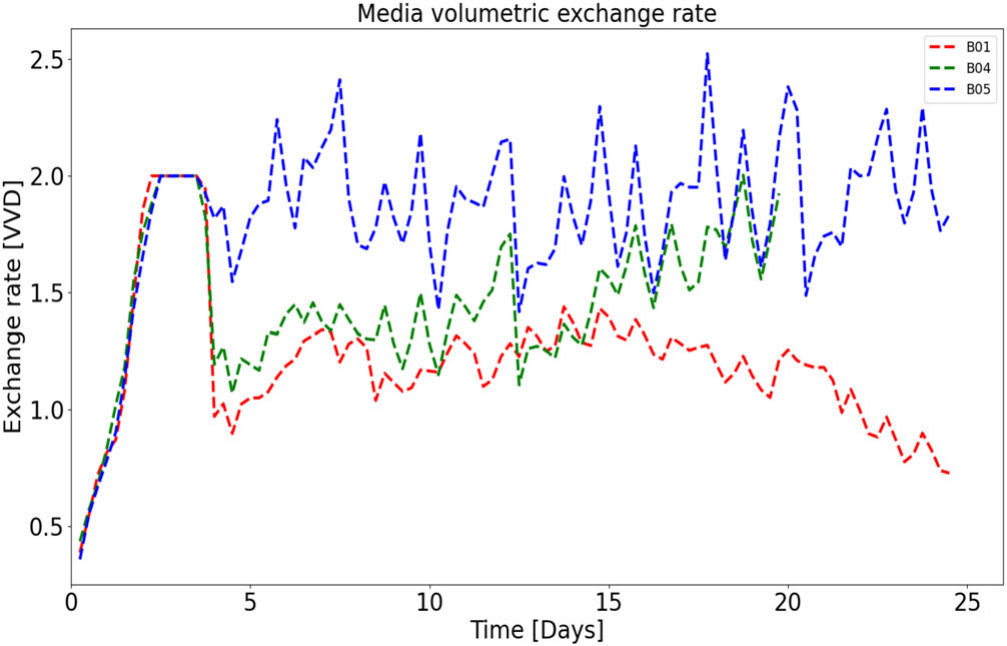
Three representative runs are shown with high, low, and in‐between media exchange rates (~2, ~1, and 1–2 vessel volumes per day [VVD]).

**FIGURE 4:**
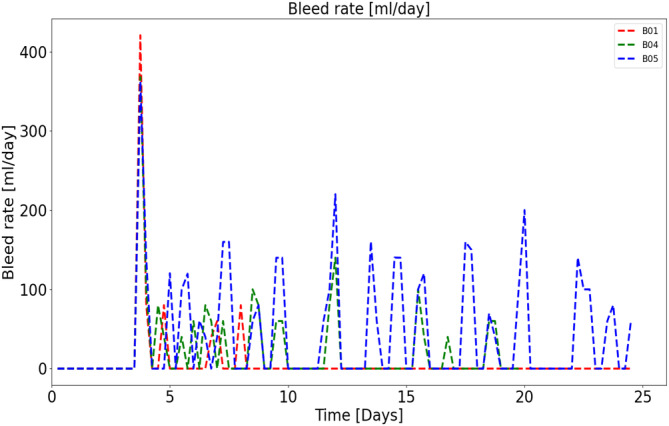
Bleed rates for perfusion runs with high, low, and in‐between media exchange rates (~2, ~1, and 1–2 vessel volumes per day [VVD]).

Various feeding strategies were performed to observe the cell response. In general, perfusion rates ranging from 1 to 2 volume exchanges per day were tested as shown in Figure 3. It is assumed that the inhibitory and toxic biomaterials pass through the CRD, therefore increasing the exchange rate reduces the accumulation of these biomaterials that impact growth and death rates. Further, a bleed controller is used to fit the bleed rate (shown in Figure 4) exactly while estimating optimal model parameters.

**FIGURE 5:**
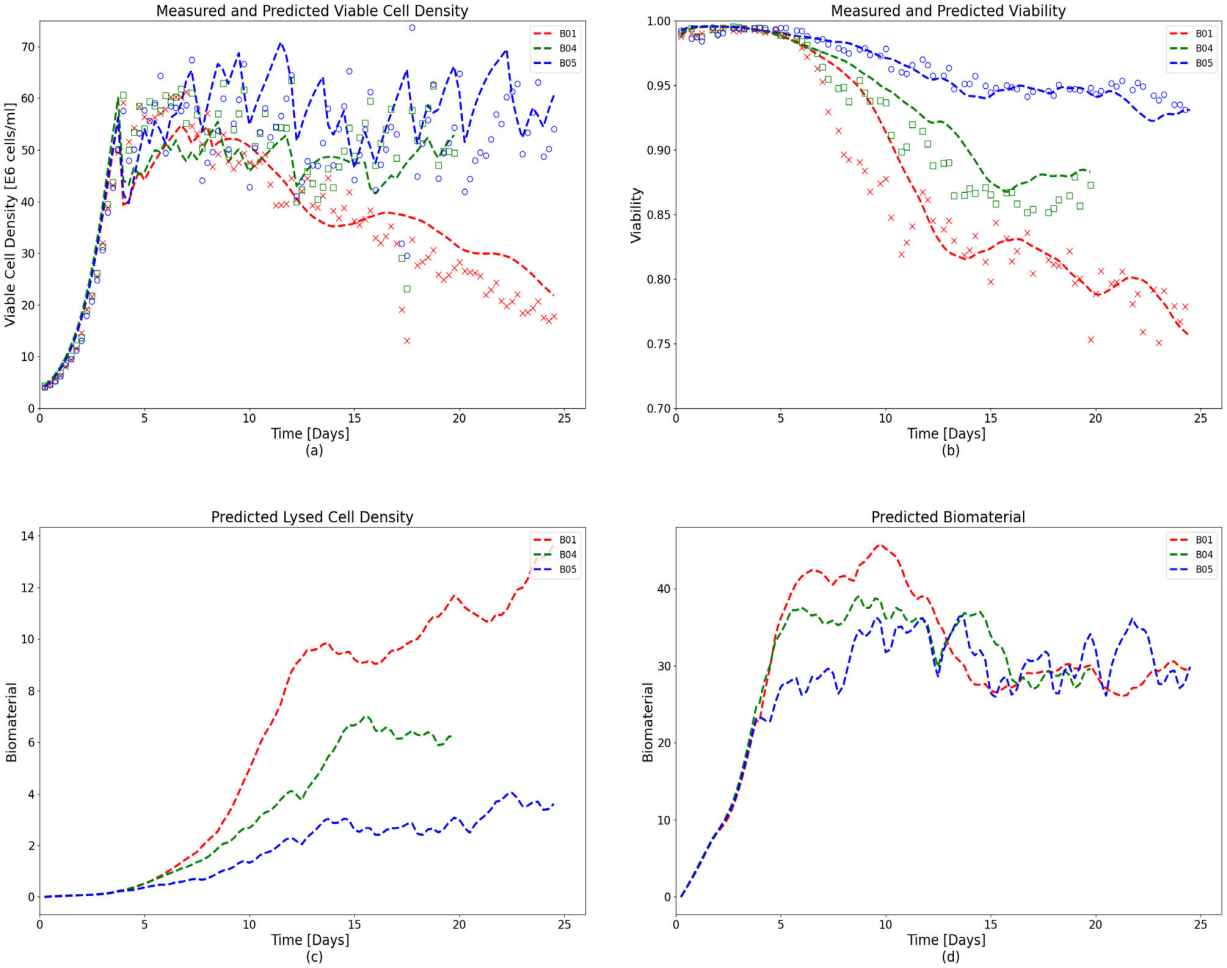
Experimental measurements (colored markers) and predicted/simulated data (dashed lines) for training batches with high, low, and in‐between media exchange rates (~2, ~1, and 1–2 vessel volumes per day [VVD]). Predicted and measured (a) viable cell density (VCD), (b) cell viability, (c) simulated lysed cell density, and (d) bio‐material as a function of cell culture time.

Cell cultures with different perfusion rates were fitted using the abovementioned method. These runs were inoculated with a cell density of 2.5 E6 cells/mL (refer Table 1). A temperature shift from 37 to 34°C was performed when the VCD reached a value greater than 50 E6 cells/mL for the training and validation batches and greater than 60 E6 cells/mL. It was also observed that no metabolite was growth‐limiting or inhibiting evaluated using the spent media analysis. It can be seen from Figure 5 that the results showed a good match between experimental data (different color markers denoting different batches) and fitting results (dotted lines) corresponding to training batches for both the VCD and cell viability, respectively. The proposed model captures the dynamics of the entire cell culture duration. For batches B04 and B05, VCD was well controlled around 60 E6 cells/mL set‐point. However, a declining VCD trend was observed in B01. This could be attributed to the accumulation of waste or an increased toxic environment for the cells inside the bio‐reactor. While lysed cells and bio‐material cannot be measured but are included in the HSSM. The impact of inhibitory biomaterials and lysed cells on growth dynamics can be assessed by analyzing the predicted hidden states of the model. Plots of hidden variables from the hybrid state‐space model, that is, bio‐material and lysed cell density, are shown in Figure 5. It is observed that accumulation of biomaterials is inhibitory to growth, impacts cell health and performance and lysed cells increase the death rate in the cell culture. It can be seen from Figure 5c that lysed cell concentration for batch B01 is extremely high as compared to other training batches thus contributing to the declining VCD trend.

**FIGURE 6:**
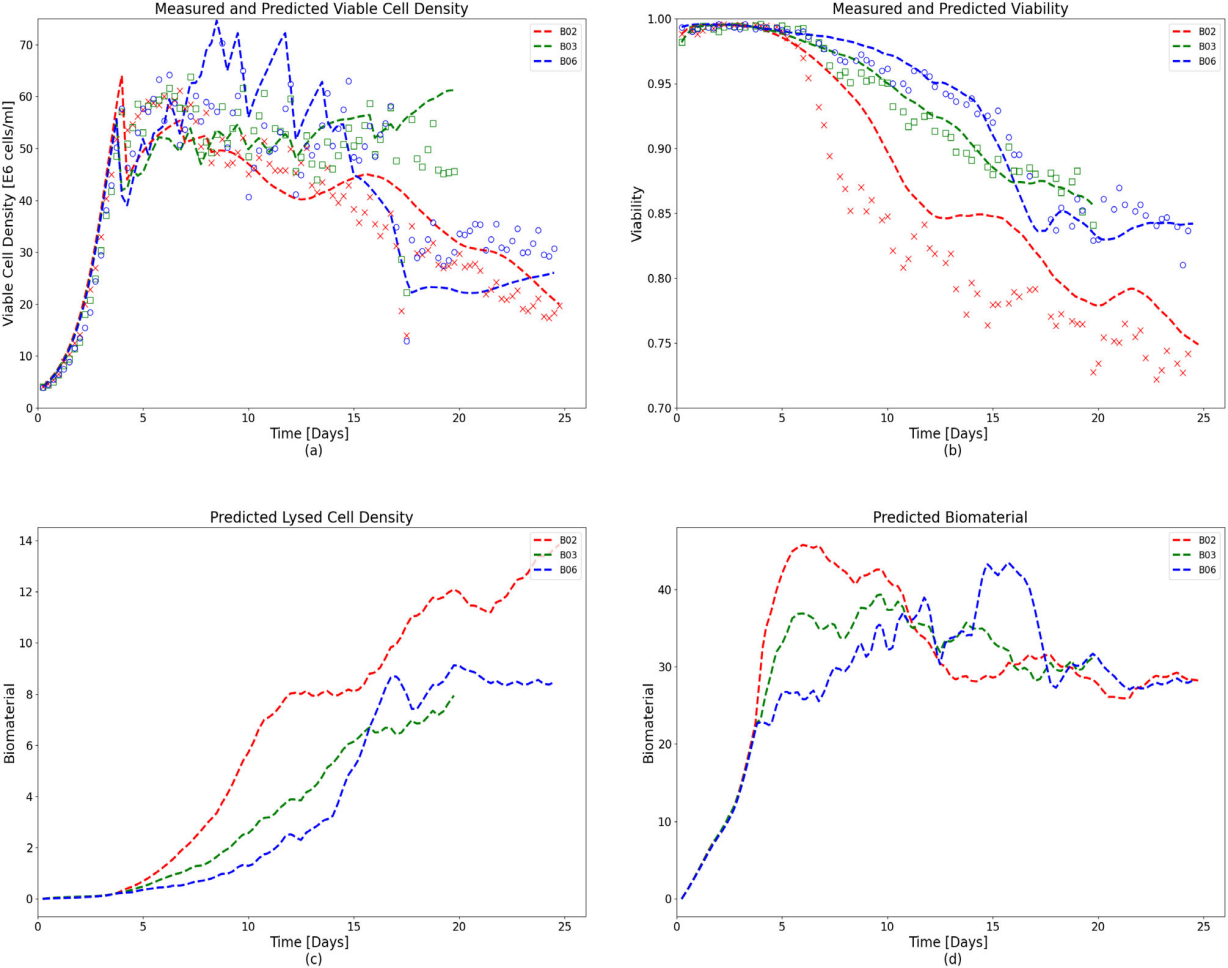
Experimental measurements (colored markers) and predicted data (dashed lines) for validation batches with high, low, and in‐between media exchange rates (~2, ~1, and 1–2 vessel volumes per day [VVD]). Fitted and measured (a) viable cell density (VCD), (b) cell viability, (c) simulated lysed cell density, and (d) bio‐material as a function of cell culture time.

**FIGURE 7:**
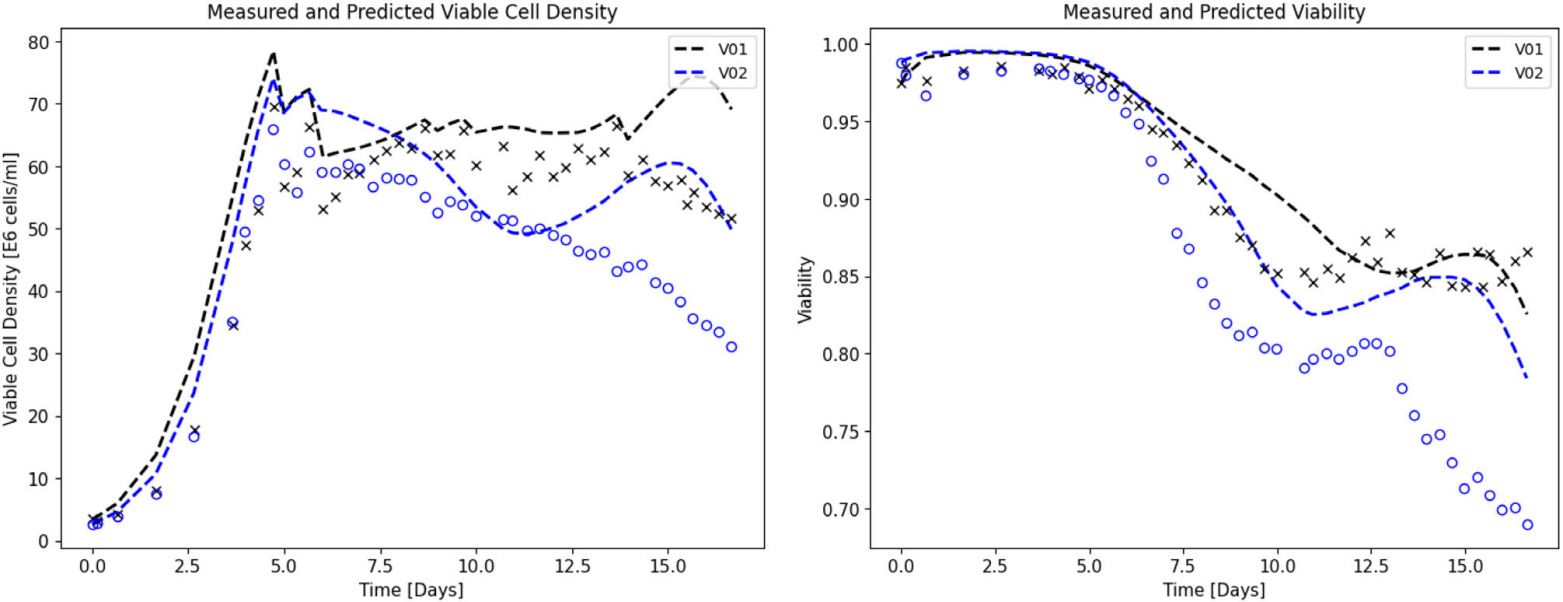
Experimental measurements (colored markers) and predicted/simulated data (dashed lines) for batches V01 and V02 media exchange rates.

The growth model was cross‐validated using data from other Ambr® 250 perfusion cultures (batches: B02, B03, and B06). The model simulations successfully predicted the culture dynamics for validation data with different operating conditions as shown in Figure 6. Two more Ambr runs (batches: V01 and V02) were carried out with operating conditions outside of the training set to further validate the modeling approach. The Ambr vessels were inoculated at 25 E5 cells/mL seeding density with a targeted production cell density of 65 E6 cells/mL. The growth model simulations successfully predicted the culture dynamics for both the batches (batches: V01 and V02) with different operating conditions (refer to Table 2) as shown in Figure 7. Subsequently, a productivity model is calibrated using the same training data and predictions from the validated growth model and is presented in the next section.

**FIGURE 8:**
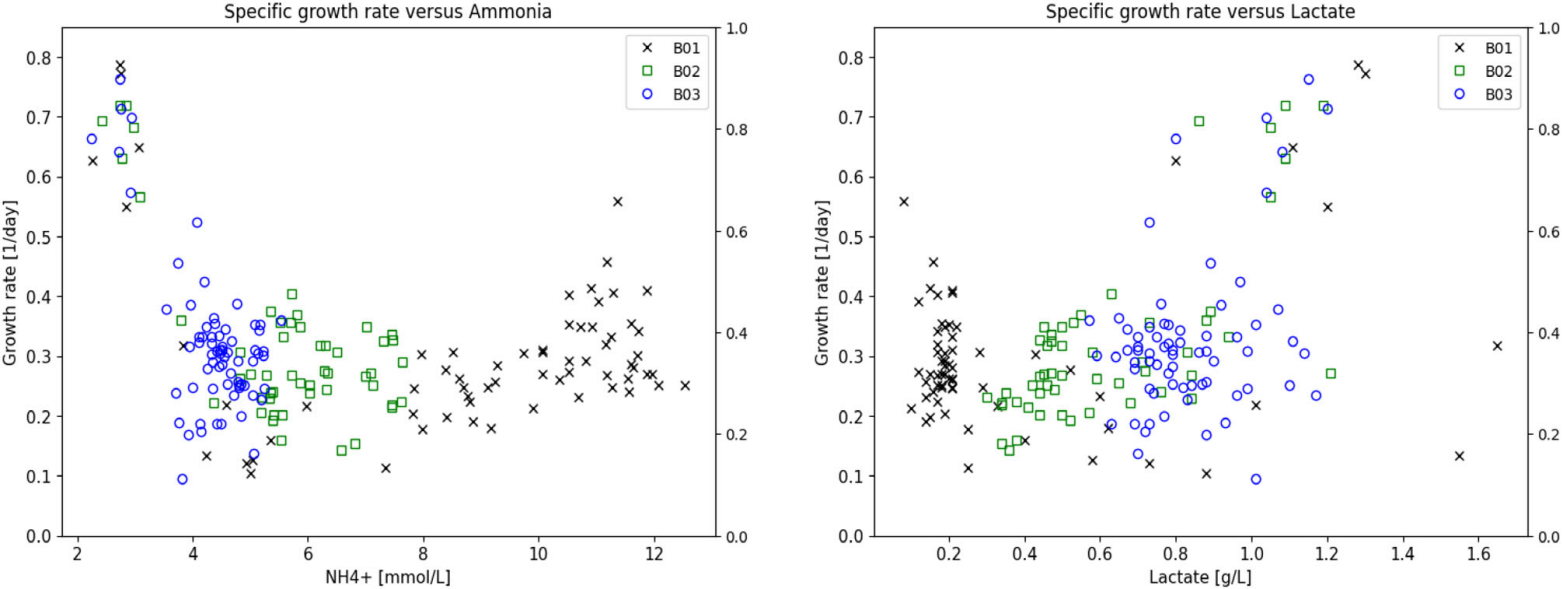
Plots of observed specific growth rate with Ammonia and Lactate, demonstrating the lack of correlation between these metabolites and growth rate.

**FIGURE 9:**
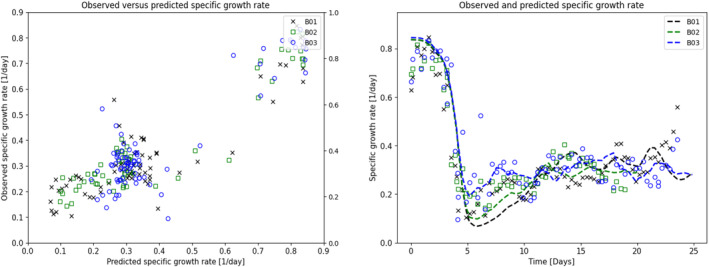
Plots of observed versus predicted specific growth rate predicted from Equation (7) and the time trajectory of the observed and predicted specific growth rate.

**FIGURE 10:**
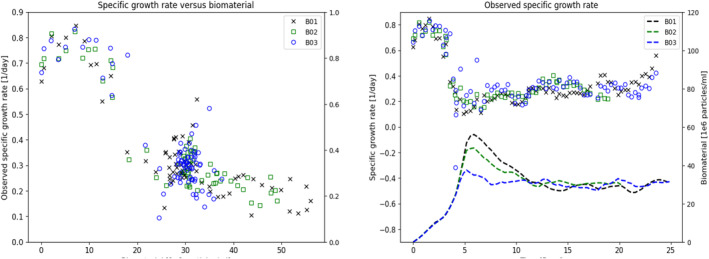
Plots of observed specific growth rate versus predicted biomaterial ($\Phi_{b}$) and the time trajectory of the observed specific growth rate and biomaterial.

Ammonia and lactate were evaluated as potential growth‐inhibiting compounds. Figure 8 shows the lack of correlation between the observed specific growth rate versus metabolite concentrations. This is in contrast to the specific growth rate predicted from the growth rate function, Equation (7), as shown in Figure 9 where there is a very good relationship between observed and predicted growth rates. The main driver of the variation in specific growth rate is the accumulation of biomaterials as shown in Figure 10.

**FIGURE 11:**
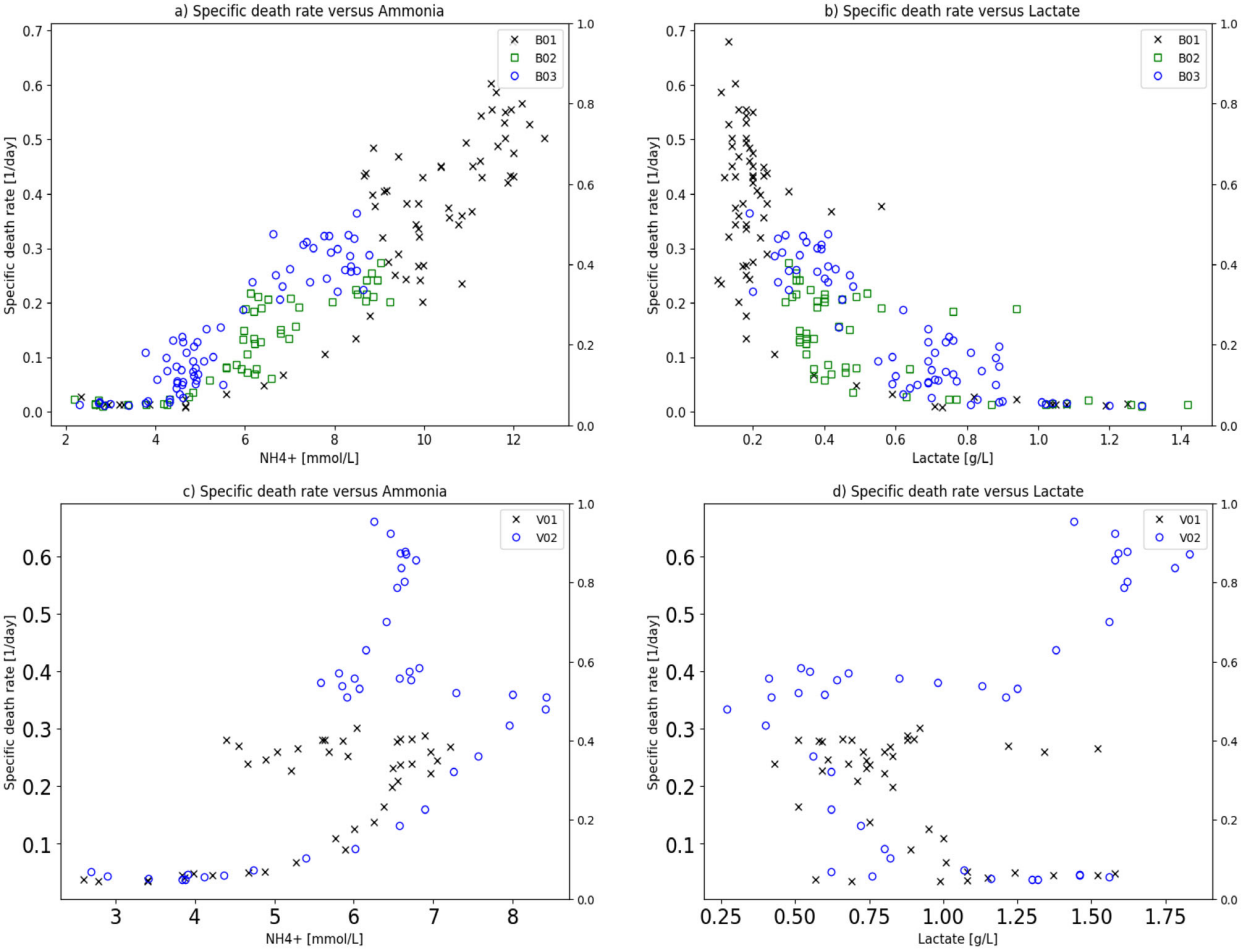
Plots of (a) observed specific death rate versus ammonia, (b) observed specific death rate versus lactate, (c) observed specific death rate versus ammonia for a later manufacturing campaign, and (d) observed specific death rate versus lactate for a later manufacturing campaign.

Similar to the modeling of the growth rate, the impact of measured metabolites on specific death rate was also evaluated. In this case, the potential of ammonia to explain elevated death rate appeared to be promising as shown in Figure 11. In validation against a set of data from a later manufacturing campaign where the recipe had been modified the relationship between ammonia and the specific death rate was no longer observed as shown in Figure 11.

**FIGURE 12:**
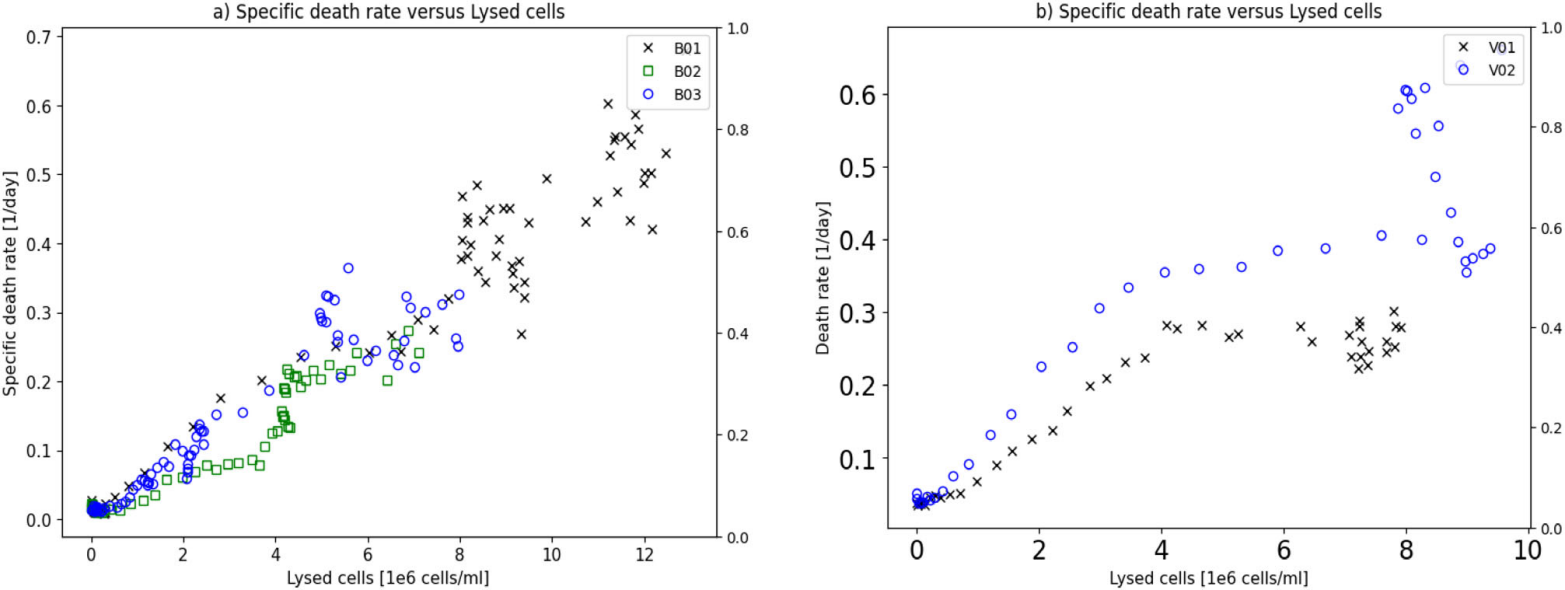
Plots of (a) observed specific death rate with predicted lysed cells and, (b) observed specific death rate with predicted lysed cells for a later manufacturing campaign.

Figure 12 shows the relationship between the observed specific death rate versus the predicted lysed cell concentration for both the original training set and also the later manufacturing campaign showing that the relationship holds well and much better than found with ammonia.

### 4.3 Benchmarking productivity model

**FIGURE 13:**
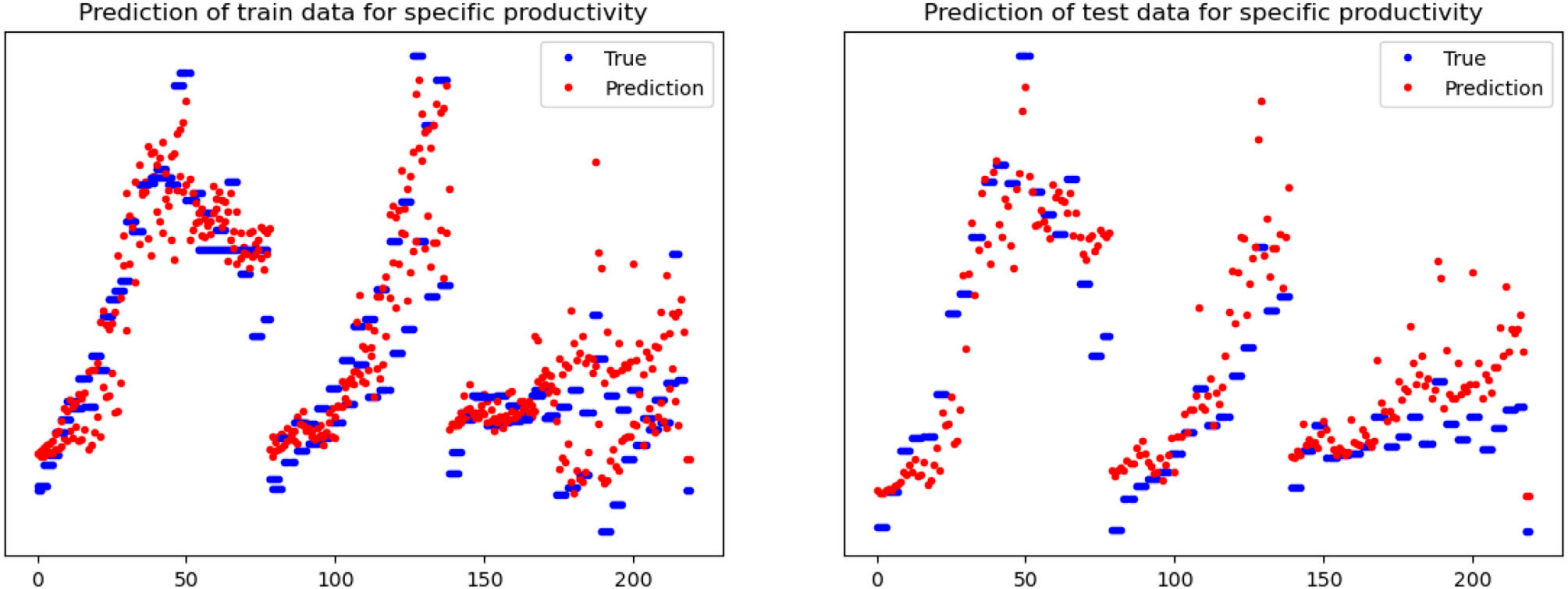
Plots of calculated and predicted specific‐productivity for training and validation batches.

**FIGURE 14:**
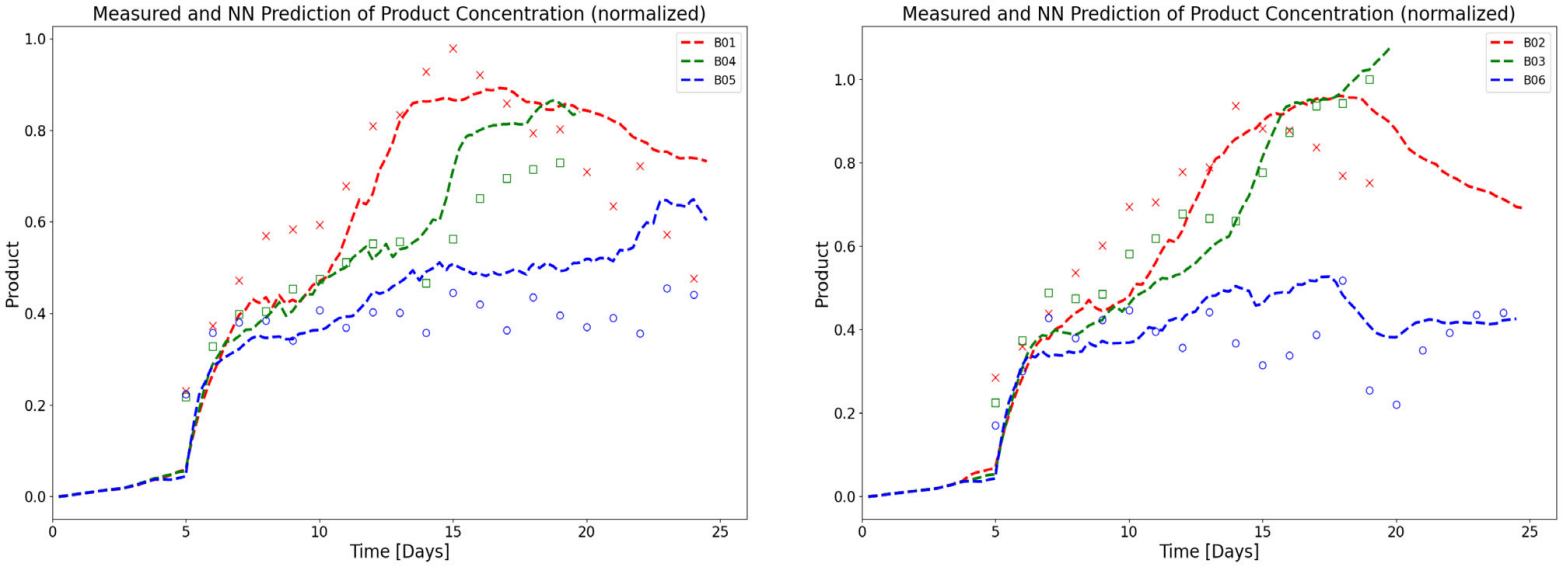
Normalized experimental measurements (colored markers) and predicted (dashed lines) titer values using a trained NN model for (a) training batches and (b) validation batches with different media exchange rates (between 1 and 2 vessel volumes per day [VVD]).

**FIGURE 15:**
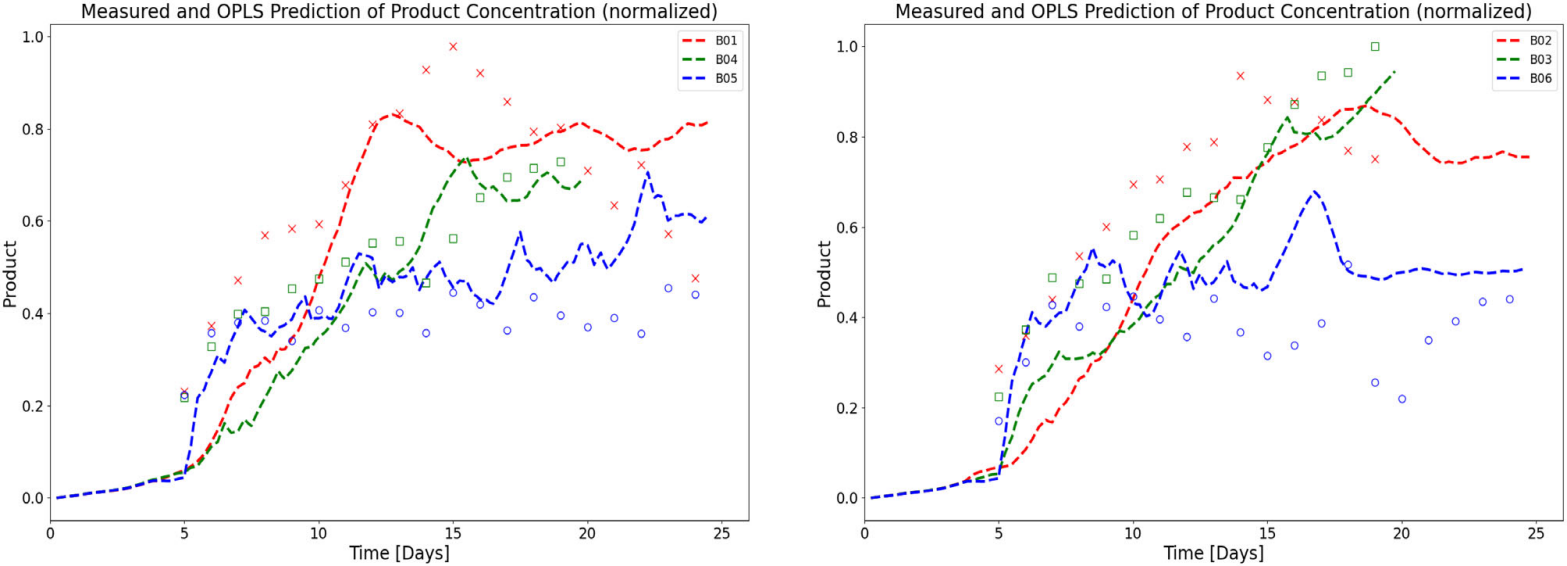
Normalized experimental data (markers) and prediction of titer (dashed lines) using the OPLS model for (a) training batches and (b) validation batches with different media exchange rates (between 1 and 2 vessel volumes per day [VVD]).

Another key objective of this work is to understand the effect of perfusion rate and different operating conditions on productivity. To this end, an OPLS model and an MLP‐NN model are trained and validated (as explained in Section 3) to predict specific productivity using process measurements and simulated hidden variables (biomaterial and lysed cell density) from HSSM. For OPLS, SIMCA is used to build the model for specific productivity and MLP‐NN is built using the Keras library in Python. A feed‐forward network of two hidden layers with 16 and 10 neurons respectively is trained with Adam optimizer. Predictions of specific productivity (refer to Figure 13) from both the models were integrated into the state equation (Equation 10) to estimate titer concentration (values are normalized and units are removed due to confidentiality). It is observed that the NN model has a lower mean square value than the OPLS model and explains the titer concentration trajectory successfully (refer to Figures 14 and 15).

**FIGURE 16:**
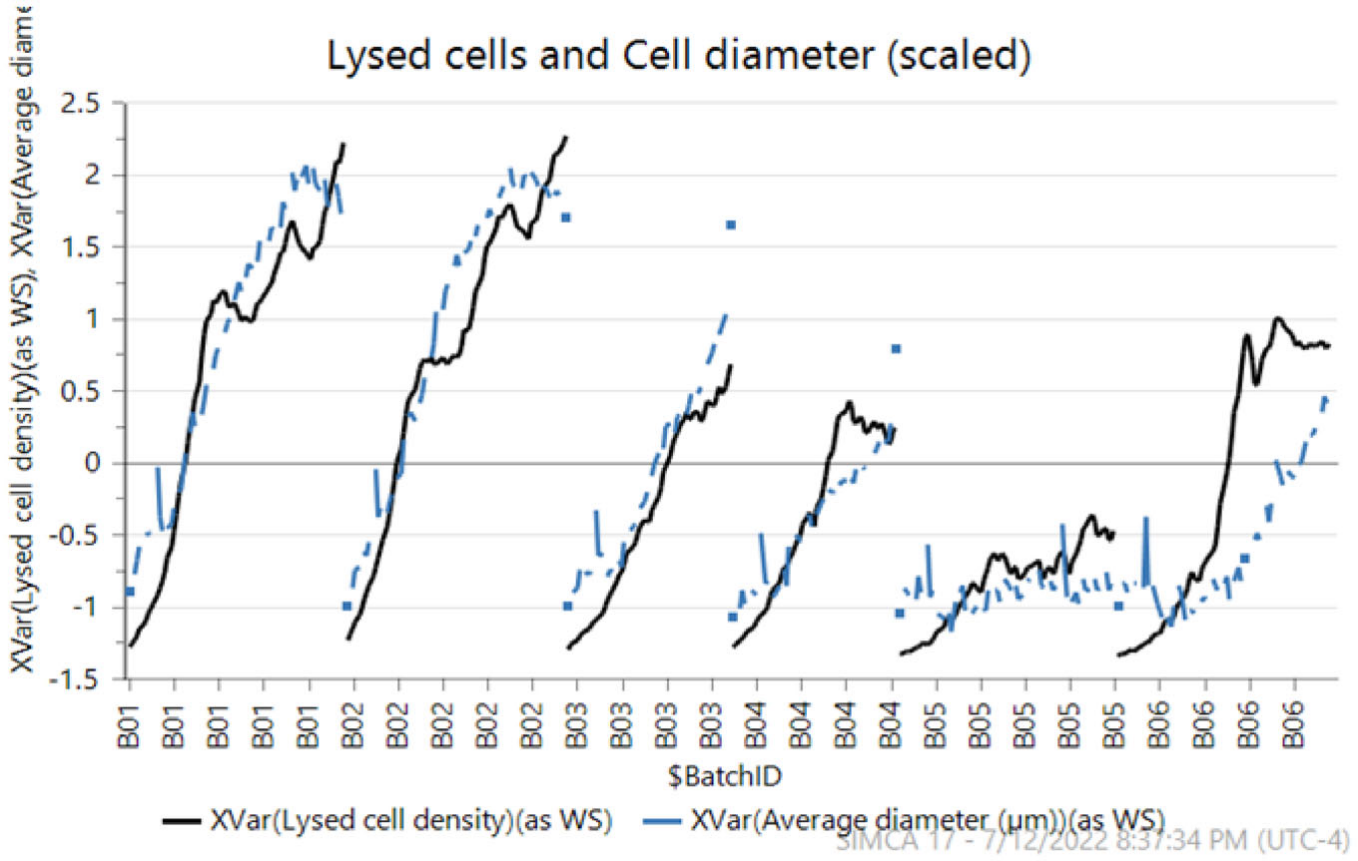
Correlation between lysed cell density and measured cell diameter.

**FIGURE 17:**
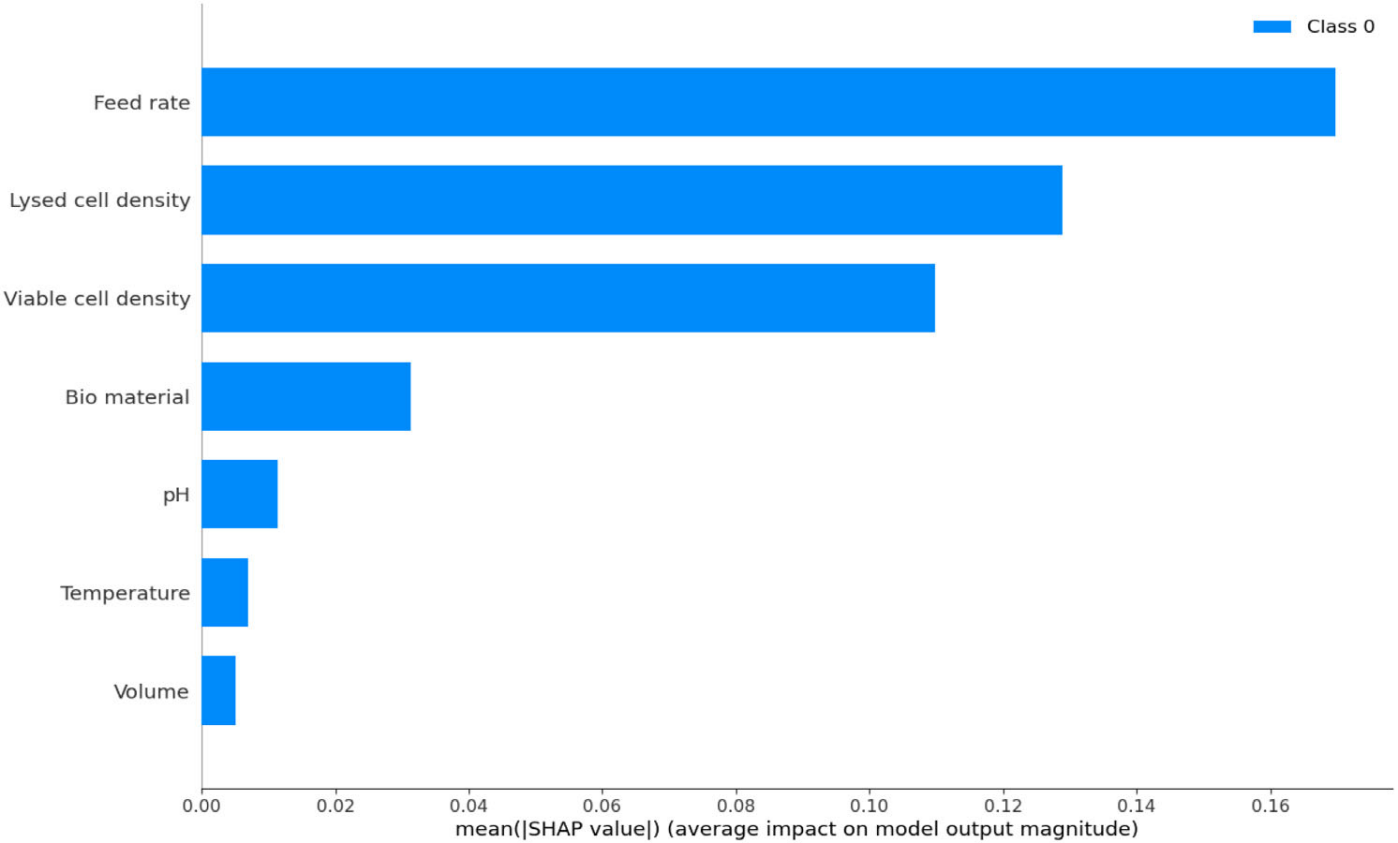
Variable importance plot (VIP) estimated using SHAP values.

It is observed from data that productivity increases at lower perfusion rates. This is linked to an increase in cell size (diameter), meaning that productivity is tied to biomass more than the number of cells, and found to be correlated to accumulation of biomaterials and lysed cells. Interestingly, cell diameter was observed to be correlated very closely to simulated lysed cell density (hidden variable in HSSM) for each batch (refer Figure 16). From the perspective of a data‐driven model, the same can be explained using concepts from eXplainable AI (XAI)^43^ that is to calculate the impact of a feature on the target variable. In this work, SHAP (SHapley Additive exPlanations) values^44^ were used to explain the NN model predictions and understand which input variable/feature is greatly influencing the specific productivity. It can be seen from the variable importance plot (refer Figure 17) estimated using SHAP that feed rate and lysed cell density have the highest influence on specific productivity. Hence, it can be concluded that the productivity model captures the biological dynamics of the cell culture process well. The process is optimized by estimating an optimal media exchange rate in the next section.

### 4.4 Process optimization

The objective of the optimization study was to determine a feeding strategy to maximize product collected in the harvest stream throughout the duration of the production phase.

(17) $$\max\int_{t_{\text{start}}}^{t_{\mathrm{end}}}\left[\mathrm{IgG}\right]F_{h}\mathrm{dt}$$

where [IgG] is the concentration of the product in the harvest stream, $t_{\text{start}}$ is the time when product collection begins and $t_{\mathrm{end}}$ is the batch completion. In the study the start and end times were set to 4 and 21 days. The optimizer selected the initial volumetric media exchange rate during intensified growth and 5 select times throughout the batch as indicated in Table 5 allowing for a dynamic yet manageable to implement feeding strategy. Also, note that once the desired viable cell density target is achieved the media exchange rate is set to 2.0 VVD with a working volume of 220 mL.

Trajectories for $\left[\mathrm{IgG}\right]$ and $F_{h}$ were simulated by running the process model with the specified exchange rates in combination with a PID feedback controller to adjust the bleed flow rate to maintain a desired viable cell density. Constant volume was assumed meaning that harvest flow is calculated as the difference between feed and bleed flow rates:

(18) $$F_{h}=F_{f}-F_{b}$$

**FIGURE 18:**
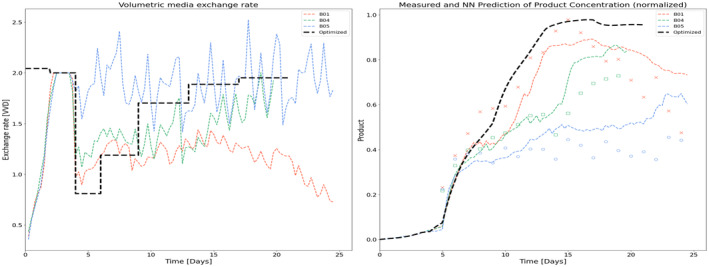
Volumetric media exchange rates and product concentration for reference and optimized batch trajectories.

The optimizer selected the following volumetric media exchange rates in Table 5 to maximize the product collected. Figure 18 shows these exchange rates and the predicted product concentration in comparison to batches in the training set for context and comparison.

**FIGURE 19:**
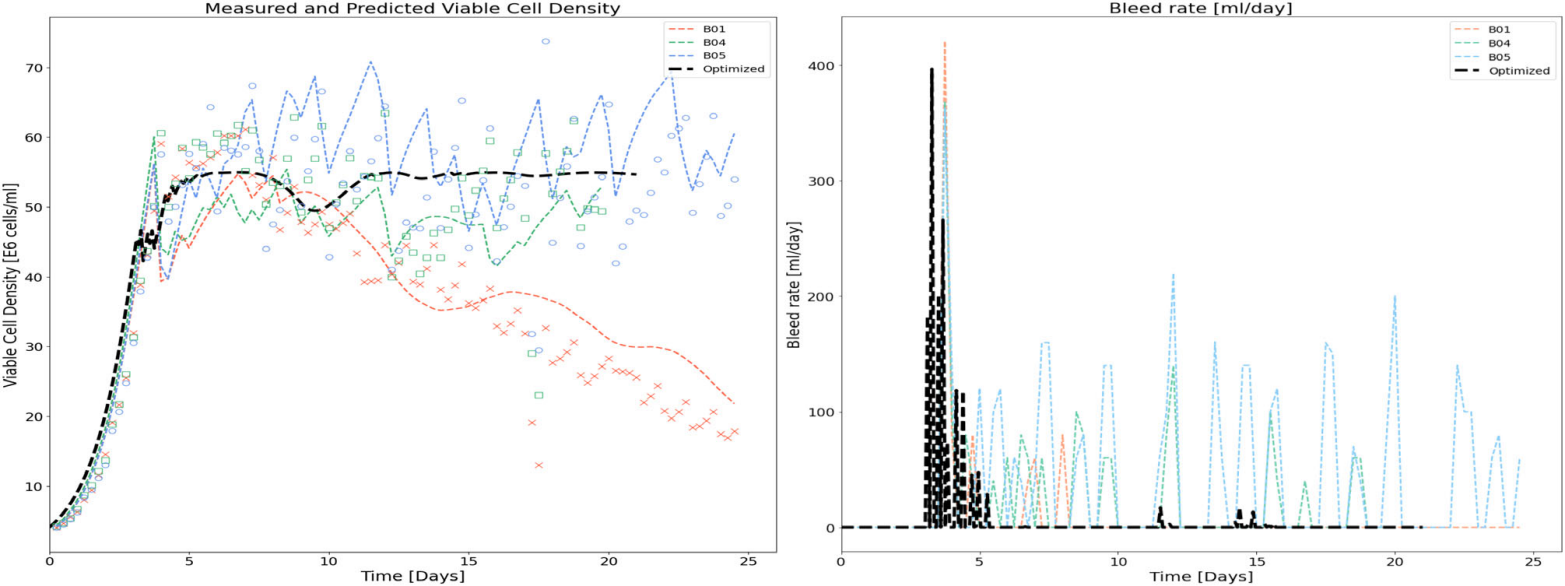
Trajectories for viable cell density and bleed flow rate for optimized and reference batches.

**FIGURE 20:**
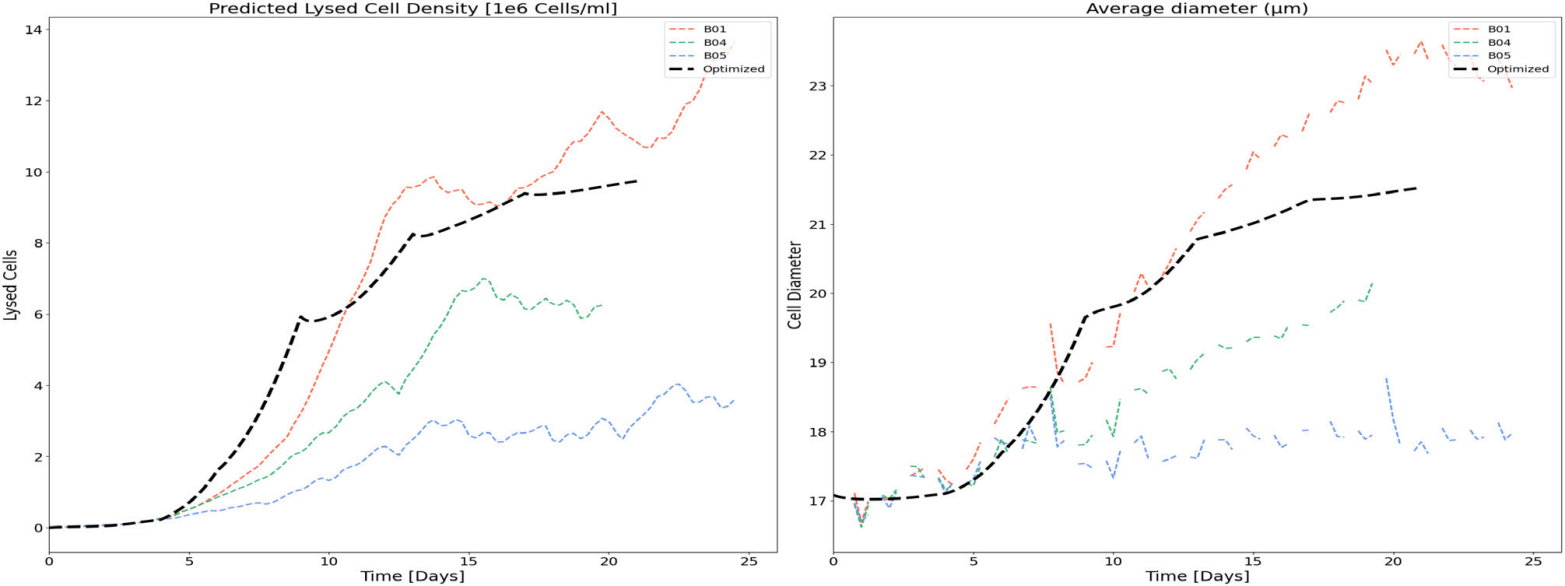
Trajectories for lysed cell density and diameter for optimized and reference batches.

**FIGURE 21:**
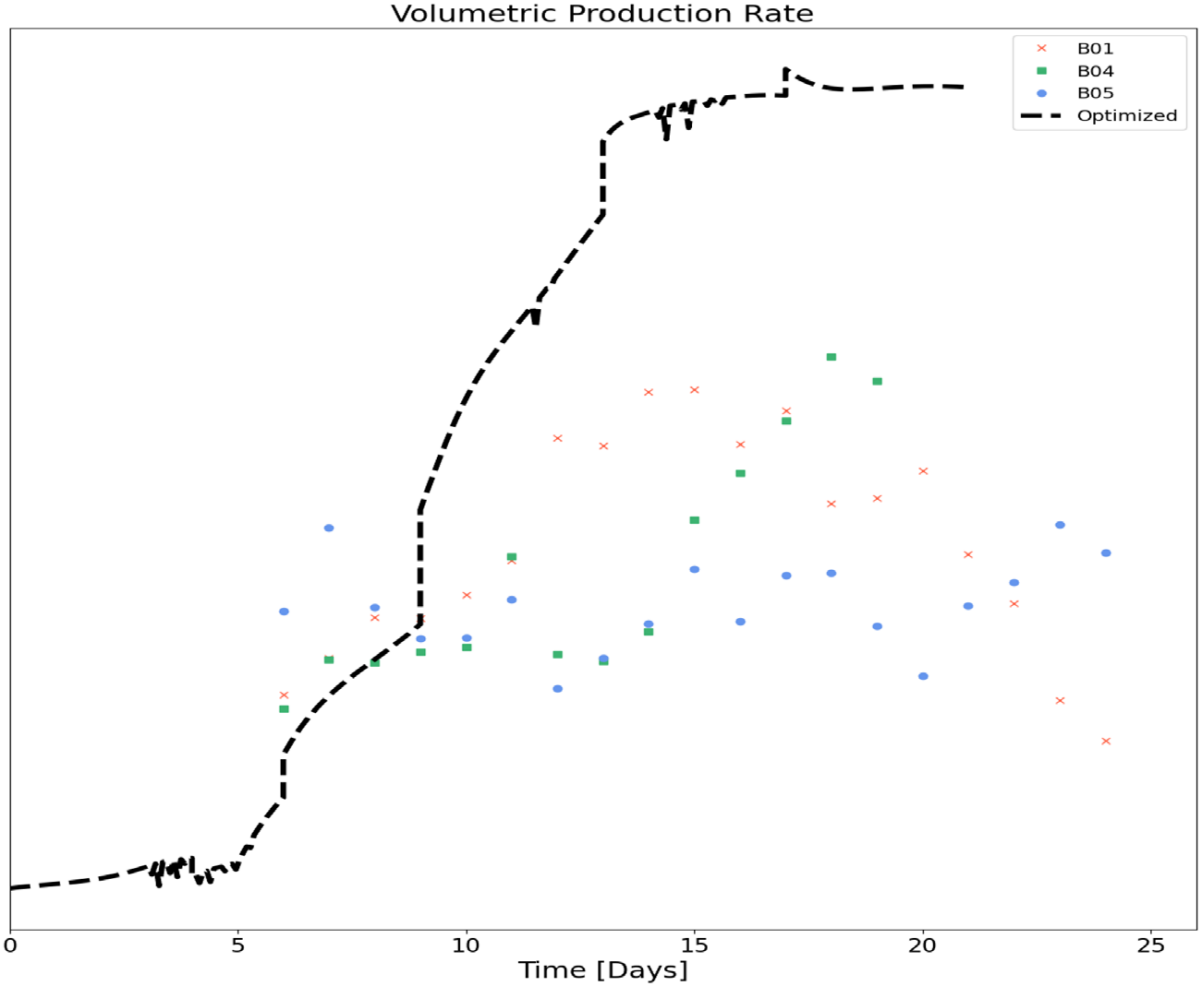
Volumetric productivity for optimized and reference batches.

The optimized feeding strategy results in a trajectory that, following the growth phase, initially reduces the exchange rate to accumulate lysed cells and biomaterial which in turn results in increased biomass and specific productivity. Figures 19 and 20 show the resulting viable cell density, bleed flow rates, and cell metrics that are accumulated lysed cells and cell diameter respectively. From these plots, we can see that the optimized feeding strategy is sufficient to maintain the target viable cell density but with minimal to no bleeding beyond the initial transition to the stationary product production phase between days 3 and 6. Minimal to no bleeding indicates that the optimizer has resulted in the lowest exchange rate to maintain cell density targets. Figure 21 shows the optimized volumetric productivity (units are hidden due to confidentiality) in comparison to the reference batches. This plot captures the overall impact of the optimized feeding strategy. A comparison of the total product captured indicates a 50% increase as compared with the best run of the training set (from Day 4 to Day 21).

**FIGURE 22:**
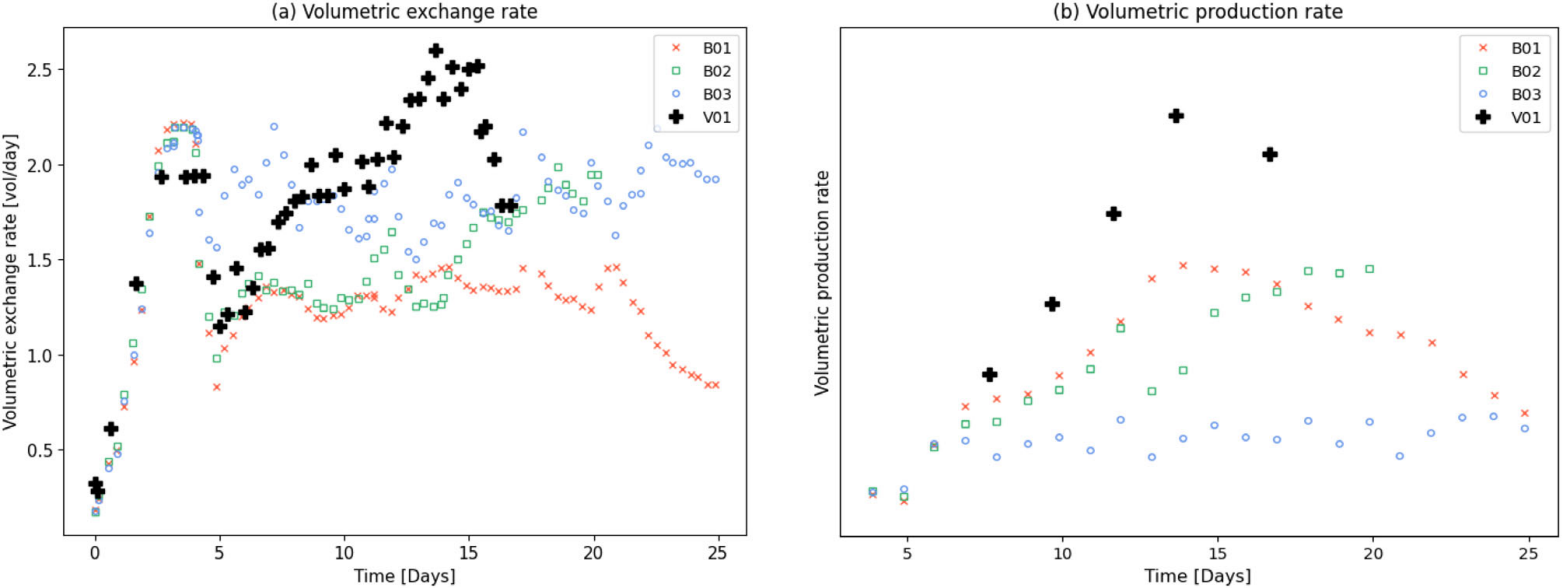
Plots of (a) media exchange rate for the training set (B01‐B03) and a later manufacturing campaign matching the optimized feeding (V01) and, (b) observed volumetric production rate.

In a follow‐up experiment (batch: V01), a feeding strategy matching the optimized feeding strategy to the ability of the control system was executed. These results verify that the recommended feeding strategy has a significant positive impact on the volumetric production rate as shown in Figure 22.

### 4.5 Scale validation: Model‐based predictions for bio‐process scale‐up

Ambr® systems have emerged as valuable tools in the bio‐pharmaceutical industry for conducting small‐scale, high‐throughput bio‐processing experiments. These miniaturized bioreactors replicate the conditions of larger bioreactors, allowing researchers to gather essential data and optimize process parameters at a smaller scale. However, the successful scale‐up of bio‐processes from Ambr® experiments to larger industrial‐scale bioreactors is a critical step that comes with its own set of challenges and considerations.

One of the goals of conducting Ambr® 250 experiments is to generate data that can be reliably extrapolated to larger bioreactors. However, ensuring the transferability of results requires careful evaluation and understanding of the differences between the Ambr® system and the target large‐scale bioreactor. Factors such as agitation, aeration, mixing, and mass transfer rates may vary between the two systems, potentially affecting the performance of the bio‐process. It is essential to account for these differences and validate the scalability of the process to ensure consistent results. Toward this end, variables such as temperature, pH, feed composition, feed rates, and media exchange rates are treated as independent variables and defined as inputs. As such, there is no thermodynamic model to represent changes in temperature or models of aeration, base addition and other influences of pH. It is assumed the regulatory control layer provides a controllable process that maintains inputs at their set‐points. This decision is taken to remove scale and hardware‐dependent dynamics, producing a scale‐independent model. This also means that the HSSM model is primarily relevant for processes which have established stable regulatory control. Given the use cases targeted for this model center around optimization and system configuration, the separation of scale‐dependent and regulatory control issues limits the scope of the HSSM to be manageable and fit for purpose.

**FIGURE 23:**
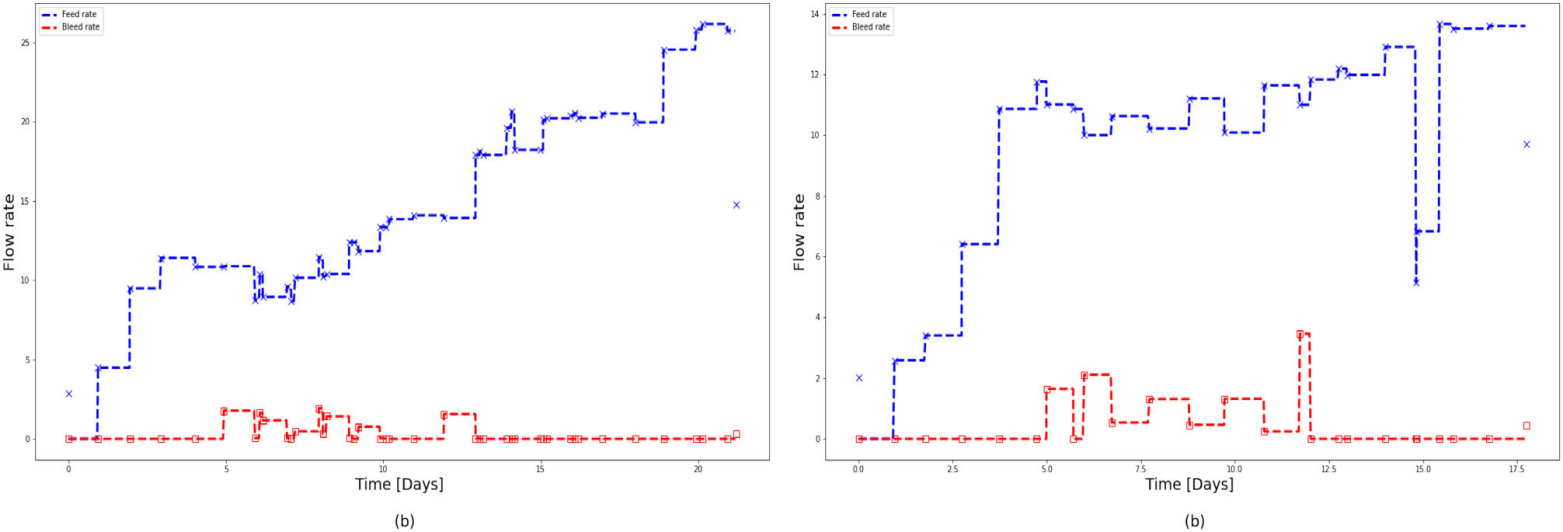
Feed rate and bleed rate for Run 1 and Run 2.

**FIGURE 24:**
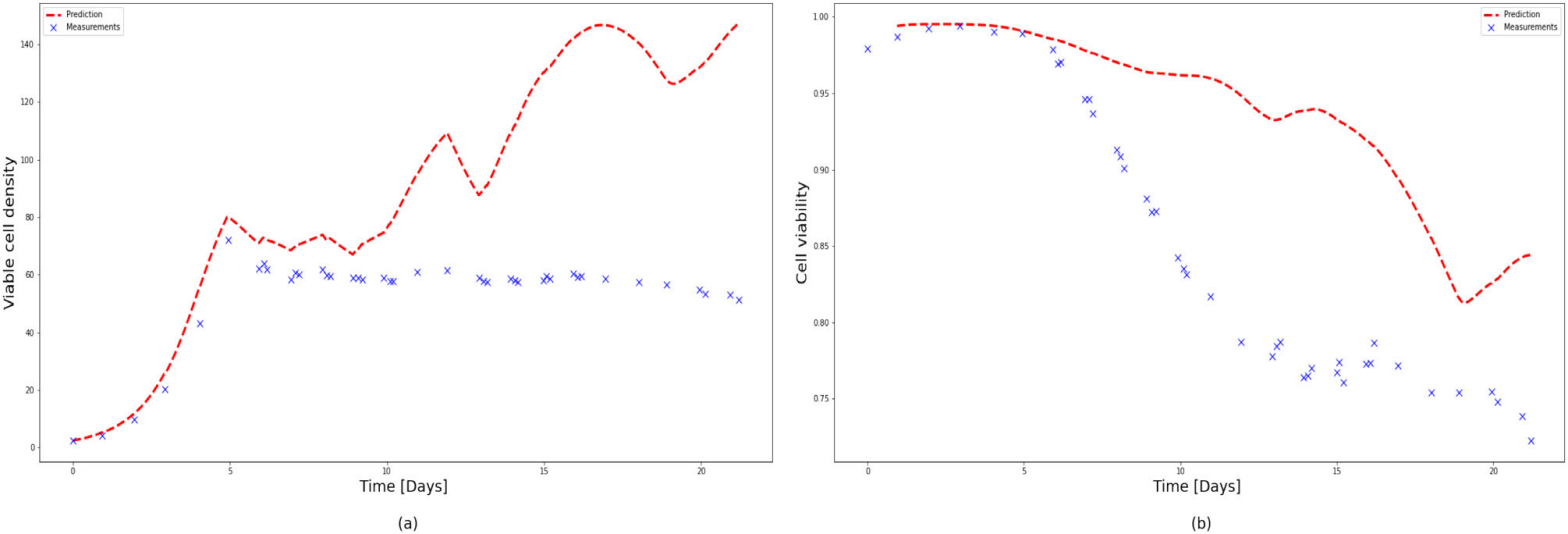
Experimental measurements (colored markers) and predicted/simulated data (dashed lines) for 5 L scale perfusion run (Run 1). Predicted and measured (a) viable cell density (VCD), (b) cell viability, as a function of cell culture time.

**FIGURE 25:**
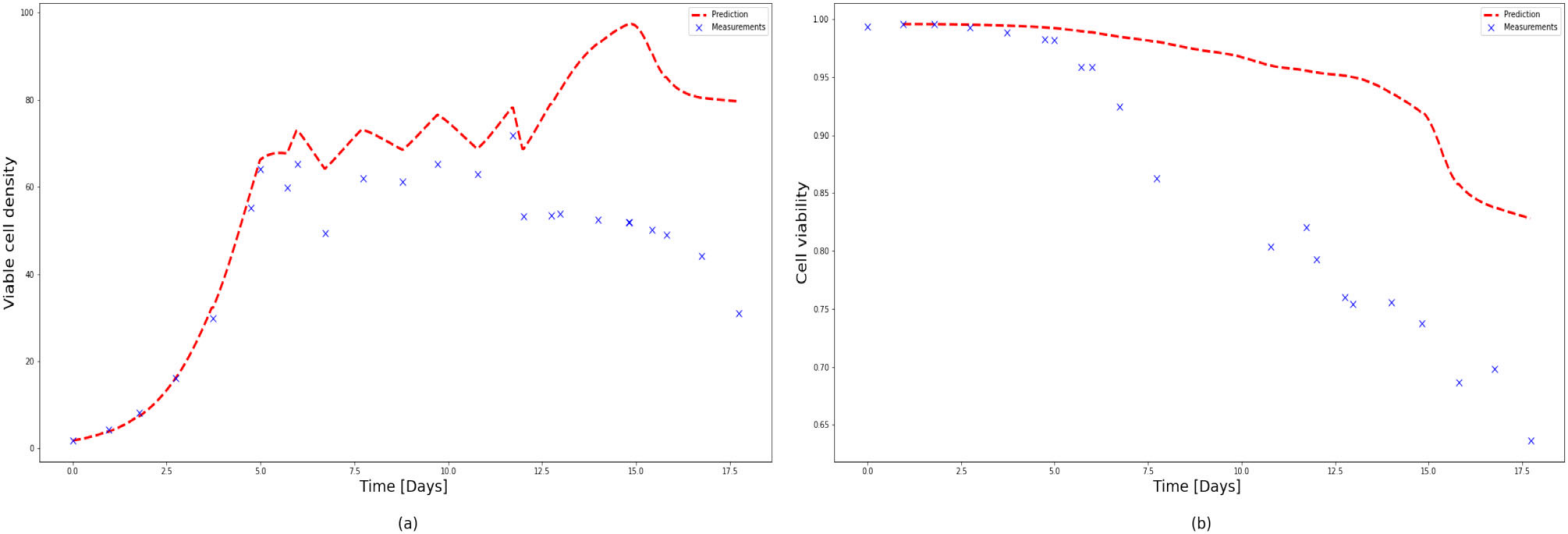
Experimental measurements (colored markers) and predicted/simulated data (dashed lines) for 5 L scale perfusion run (Run 2). Predicted and measured (a) viable cell density (VCD), (b) cell viability, as a function of cell culture time.

To test the extrapolation capability of HSSM identified from Ambr 250 mL experiments, 5 L perfusion runs (Run 1 and Run 2) were simulated using the identified model (refer to Sections 4.1 and 4.2) with different feeding strategies as shown in Figure 23. It is observed that VCD and viability predictions for both runs deviate significantly after 12.5 days as shown in Figures 24 and 25. It was hypothesized the difference in growth dynamics could be explained by a sieving effect in the CRD with respect to biomaterials and lysed cells at the 5 L scale. It was also confirmed by BiosanaPharma internally that there are differences in the CRD filter membrane types, lengths, and area per volume used for Ambr® and 5 L benchtop bioreactor. To test this hypothesis, the lysed cells and biomaterials were split into two parts, consisting of those that pass through the CRD and those that do not. Sieving coefficients ($k_{\mathrm{ls}}$ and $k_{\mathrm{bs}}$) relating to new state variables $X_{\mathrm{lh}}$ and $\Phi_{\mathrm{bh}}$ in the HSSM model were introduced to mimic the holdup of lysed cells and accumulated biomaterial inside the reactor as follows:

(19) $$\begin{array}{c} \frac{dX_{v}}{\mathrm{dt}}=\mu_{\mathrm{eff}}X_{v}-\mu_{d}X_{v}-\frac{F_{b}}{V}X_{v}\\ \frac{dX_{d}}{\mathrm{dt}}=\mu_{d}X_{v}-\left(k_{l}+\frac{F_{b}}{V}\right)X_{d}\\ \frac{dX_{l}}{\mathrm{dt}}=\left(1-k_{\mathrm{ls}}\right)k_{l}X_{d}-\left(\frac{F_{h}+F_{b}}{V}\right)X_{l}\\ \frac{dX_{\mathrm{lh}}}{\mathrm{dt}}=k_{\mathrm{ls}}k_{l}X_{d}-\left(\frac{F_{h}+F_{b}}{V}\right)X_{\mathrm{lh}}\\ \frac{d\Phi_{b}}{\mathrm{dt}}=\left(1-k_{\mathrm{bh}}\right)X_{v}-\left(\frac{F_{h}+F_{b}}{V}\right)\Phi_{b}\\ \frac{d\Phi_{\mathrm{bh}}}{\mathrm{dt}}=k_{\mathrm{bh}}X_{v}-\left(\frac{F_{h}+F_{b}}{V}\right)\Phi_{\mathrm{bh}} \end{array}$$

**FIGURE 26:**
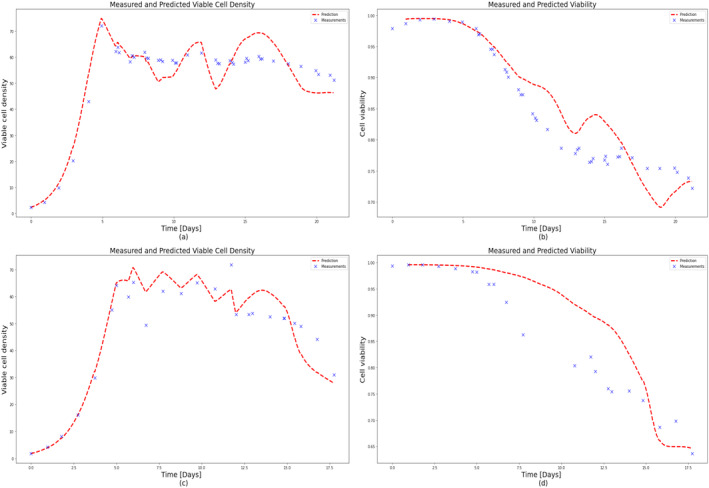
Experimental measurements (colored markers) and predicted/simulated data (dashed lines) for both 5 L scale perfusion runs (Run 1 and Run 2). Predicted and measured (a) Run 1: viable cell density (VCD), (b) Run 1: cell viability, (c) Run 2: VCD, (d) Run 2: cell viability, as a function of cell culture time.

It should be noted that parameters such as maximum growth rate: $\mu_{\max}$, specific death rate: $\mu_{d}$, lysing rate: $k_{l}$ and spread coefficients for the quadratic effect of inputs, that is, pH and temperature: $\theta_{q,\mathrm{pH}}$ and $\theta_{q,\text{temp}.}$ are kept the same as identified from Ambr® experiments previously and only toxicity rate: $k_{t}$, sieving coefficients $k_{\mathrm{ls}}$, $k_{\mathrm{bh}}$, and inhibition coefficient $K_{I,\Phi_{b}}$ are estimated by using an optimizer fitting VCD and cell viability. Additionally, note that inhibition from accumulated biomaterials is now modeled as the sum of the two biomaterials (biomaterial from Equation 5 is equal to the sum of the two biomaterial states) and similarly the acceleration of the death rate is now explained by the sum of the two lysed cell states. Optimal values of these parameters are presented in Table 6 and plots of predicted VCD and cell viability in Figure 26 for both 5 L perfusion runs.

## 5 CONCLUSIONS

The development and calibration of the hybrid model for simulating growth, cell health, and specific productivity mark a significant advance in understanding the underlying drivers of cell performance. The developed model, in this work, not only supports in silico experimentation—allowing digital testing of new feeding and optimization strategies before laboratory validation—but also offers insights into growth trajectories and the impact of different media exchange modes. By using data from Ambr® 250 perfusion experiments, the model successfully predicted growth profiles under various feeding conditions, demonstrating its effectiveness in evaluating new strategies and reducing the need for extensive wet lab experiments.

Additionally, the implementation of an optimization algorithm showed a 50% increase in product yield, validated through a dynamic media exchange rate schedule that balanced productivity with the effects of accumulated lysed cells and biomaterials. This hybrid model provides a reliable alternative for process optimization to traditional experimental methods, which often require exhaustive DoE approaches and may not easily handle simultaneous variations in multiple control variables. We also presented successful simulations of 5 L perfusion runs using a model identified based on Ambr® experiments demonstrating reliable extrapolation capabilities of the hybrid model to large bioreactors.

With continued experimentation and data collection, this versatile model can be further refined to include additional metabolic information, such as changes in pH, temperature, and media composition. Ultimately, this model can be used to optimize media composition and make informed decisions to enhance productivity while maintaining Critical Quality Attributes within desired parameters.

## AUTHOR CONTRIBUTIONS

**Piyush Agarwal:** Conceptualization; formal analysis; investigation; methodology; software; writing—original draft. **Chris McCready:** Conceptualization; data curation; investigation; software; supervision; writing—original draft. **Say Kong Ng:** Investigation. **Jake Chng Ng:** Investigation. **Jeroen van de Laar:** Investigation. **Maarten Pennings:** Project administration; resources; supervision. **Gerben Zijlstra:** Conceptualization; project administration; supervision.

## CONFLICT OF INTEREST STATEMENT

Piyush Agarwal (at the time of manuscript submission), Chris McCready, and Gerben Zijlstra were Sartorius employees. Maarten Pennings, Jeroen van de Laar, and Jake Chng Ng were BiosanaPharma employees and Say Kong Ng was BTI employee. The authors declare no conflict of interest.

### PEER REVIEW

The peer review history for this article is available at https://www.webofscience.com/api/gateway/wos/peer-review/10.1002/btpr.3503.

## Data Availability

The data that support the findings of this study are available from the corresponding author upon reasonable request.
